# The lasting after-effects of an ancient polyploidy on the genomes of teleosts

**DOI:** 10.1371/journal.pone.0231356

**Published:** 2020-04-16

**Authors:** Gavin C. Conant

**Affiliations:** 1 Department of Biological Sciences, North Carolina State University, Raleigh, NC, United States of America; 2 Bioinformatics Research Center, North Carolina State University, Raleigh, NC, United States of America; 3 Program in Genetics, North Carolina State University, Raleigh, NC, United States of America; 4 Division of Animal Sciences, University of Missouri, Columbia, MO, United States of America; Universite de Lausanne Faculte de biologie et medecine, SWITZERLAND

## Abstract

The ancestor of most teleost fishes underwent a whole-genome duplication event three hundred million years ago. Despite its antiquity, the effects of this event are evident both in the structure of teleost genomes and in how the surviving duplicated genes still operate to drive form and function. I inferred a set of shared syntenic regions that survive from the teleost genome duplication (TGD) using eight teleost genomes and the outgroup gar genome (which lacks the TGD). I then phylogenetically modeled the TGD’s resolution via shared and independent gene losses and applied a new simulation-based statistical test for the presence of bias toward the preservation of genes from one parental subgenome. On the basis of that test, I argue that the TGD was likely an allopolyploidy. I find that duplicate genes surviving from this duplication in zebrafish are less likely to function in early embryo development than are genes that have returned to single copy at some point in this species’ history. The tissues these ohnologs are expressed in, as well as their biological functions, lend support to recent suggestions that the TGD was the source of a morphological innovation in the structure of the teleost retina. Surviving duplicates also appear less likely to be essential than singletons, despite the fact that their single-copy orthologs in mouse are no less essential than other genes.

## Introduction

The study of doubled genomes (or polyploids) has a long history in genetics [1–5], but it was the advent of complete genome sequencing that most dramatically confirmed the role of polyploidy in shaping eukaryote genomes [6]. The remnants of ancient genome duplications have been found across the eukaryotic phylogeny, from plants [7] and yeasts [8] to ciliates [9], vertebrates [5, 10, 11], nematodes [12] and arachnids [13].

Flowering plants may be the “champions” of polyploidy [7], but genome duplication has also extensively shaped the evolution of teleost fishes [14–17]. Events ranging in age from recent (<1Mya) hybridization-induced polyploidies to very old genome duplications are known, including events shared among clades in the salmonids, carps, and sturgeons. The event considered here occurred between 320 and 400 Mya in the ancestor of most ray-fined fishes: the *teleost genome duplication* [TGD; 18, 19–22]. Evidence for this event started to accumulate in the late 1990s [14, 23, 24] and became effectively irrefutable with the sequencing of the first teleost genomes [25–27].

Evolutionary changes associated with the TGD include divergence in vitamin receptors [28], circulatory system genes [29] and in the structure of core metabolism [30]. Indeed, the classic example of duplicate gene divergence by subfunctionalization involves two zebrafish *ohnologs* [duplicates that are the products of a WGD; 31] from the TGD: *eng1a* and *eng1b* [32]. At the genome scale, the TGD probably increased the genome rearrangement rate for a period [22], as well as increasing the rate of sequence insertions and deletions [33]. Likewise, teleost genomes show evidence for reciprocal gene losses of alternative copies of homologous genes created by the TGD, a pattern that can induce reproductive isolation between populations possessing it [34–38].

A phylogenomic study of the TGD was undertaken by Inoue and colleagues [39], who concluded that, as with other WGDs, it was followed by an initial period of very rapid duplicate gene loss [40, 41]. However, the TGD is worth revisiting, because the previous paper used gene tree/species tree reconciliation to identify its relics, an approach which has limitations relative to methods based on the analysis of blocks of double-conserved synteny [DCS; 42, 43]. For instance, Inoue et al., could not invariably phase the surviving TGD-produced duplicates into orthology relationships, making estimating loss timings more challenging. A new analysis is particularly important because zebrafish’s role as a developmental model gives us an opportunity to explore the effects of WGD on developmental evolution. That WGD’s effects may be important has long been hypothesized, with one example being the suspected role of the 2R-produced duplications of Hox genes in creating plasticity in body-plans [44].

Likewise, much of the work on the “rules” of evolution after WGD has been performed using relatively recent events, with less understanding of the very long-term effects of polyploidy. These proposed rules include the *dosage balance hypothesis* [DBH; 11, 45, 46–48]: the tendency of more highly interacting genes to remain as ohnolog pairs longer after WGD. The DBH argues that the kinetics of cellular interactions are sensitive to imbalances in the concentrations of the interacting entities [50], driving those interacting genes to be maintained in similar dosages (e.g., as ohnologs after WGD). The DBH is a powerful model because it links observations on how genomes evolve after polyploidy to other genomic patterns, such as the observed excess of detrimental effects from over-expression among genes whose products participate in protein complexes [51] and the tendency for larger relative differences in gene dosage in aneuploid organisms to give rise to larger phenotypic effects [52]. In the context of polyploidy, the effects of the DBH may not preserve ohnologs indefinitely [49], and the TGD is old enough to explore this question. A second rule of polyploidy pertains only to polyploids formed through the merging of genomes from distinct, if related species, which are known as *allopolyploids* [in contrast to autopolyploids formed from two parental genomes from the same species; 53]. In many allopolyploids *biased fractionation* is seen, whereby one of the two parental genomes retains more genes than does the other [54–60]. The role of biased fractionation in the resolution of the TGD has also not, to my knowledge, been explored.

Using POInT, the Polyploid Orthology Inference Tool [61], I modeled the resolution of the TGD using eight teleost genomes. I find that the surviving ohnologs produced by the TGD are distinct in their character even after more than 300 million years of evolution. Genes expressed in earliest phases of development lost their ohnolog partners unusually quickly after the TGD, while the surviving ohnologs are less likely to be essential in zebrafish yet occupy more central positions in its metabolic network. In addition, there are suggestions that the TGD helped shape a key innovation in the teleost visual system.

## Results

### Identifying the relics of the TGD in eight teleost genomes

**Fig 1:**
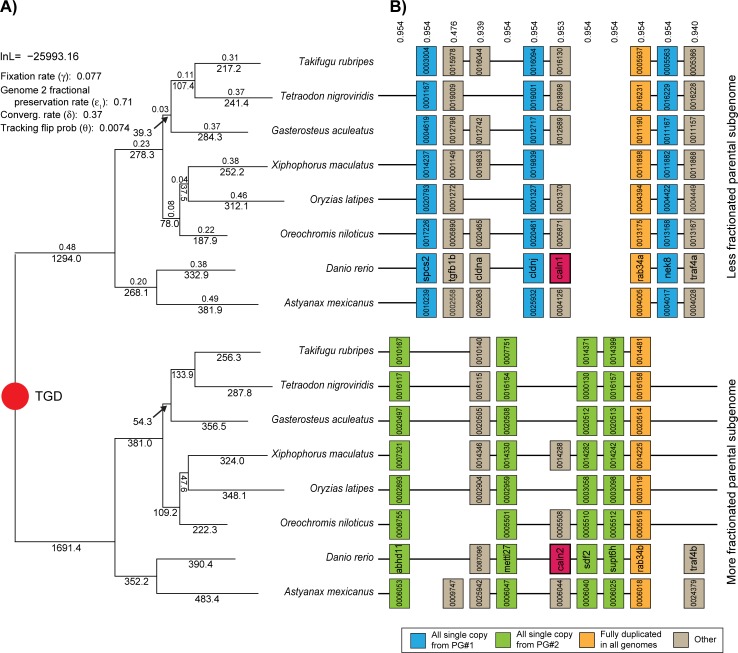
Resolution of the TGD through ohnolog losses. **A)** Shown is the assumed phylogeny of the eight species analyzed (see Methods). The TGD induces two mirrored gene trees, corresponding to the genes from the less fractionated parental genome (top) and the more fractionated parental genome (bottom, see Results for tests of the significance of the level of biased fractionation). Below the branches in each tree are POInT’s predicted number of gene losses along that branch for the parental genome in question. Above the branches in the upper tree are POInT’s branch length estimates, namely *t* (time) multiplied by the α parameter in Fig 2. Here α*t* corresponds to the overall estimated level of gene loss on that branch: a larger α*t* implies a greater number of losses *relative* to the total number of surviving ohnologs at the start of the branch. In the upper left are POInT’s parameter estimates (γ,ε_1,_δ) for the WGD-*bc*^*nbn*^*f* model (see Fig 2). **B)** An example region of the eight genomes, showing the blocks of DCS. For all species except zebrafish, truncated Ensembl gene identifiers are given; for zebrafish gene names are shown. The numbers above each column gives POInT’s confidence in the orthology relationship shown, relative to the *2*^*8*^*−1* (= 255) other possible orthology relationships. These other relationships entail swapping the two tracks of genes from one or more of the genomes between the top and the bottom panel: the confidence estimates indicate how much worse a fit is induced by assuming a different set of subgenome assignments. Genes are color-coded based on the pattern of ohnolog survival in the eight genomes. A pair of ohnologs expressed in the zebrafish retina are shown in magenta.

We have developed a pipeline [58, 62] for inferring blocks of double-conserved synteny (DCS) from a group of genomes sharing a WGD and an unduplicated reference genome (here spotted gar). This tool uses sequence similarity to identify homologous genes and then infers the products of a WGD by seeking to maximize the number of homologs that are members of such DCS blocks (*Methods*). With it, I identified 5589 loci where one or both genes from the TGD survive in all eight teleost genomes and are in synteny with at least one other locus in each genome (*Methods* and Fig 1). I refer to these loci as “pillars” [63] (c.f., Fig 1).

**Fig 2:**
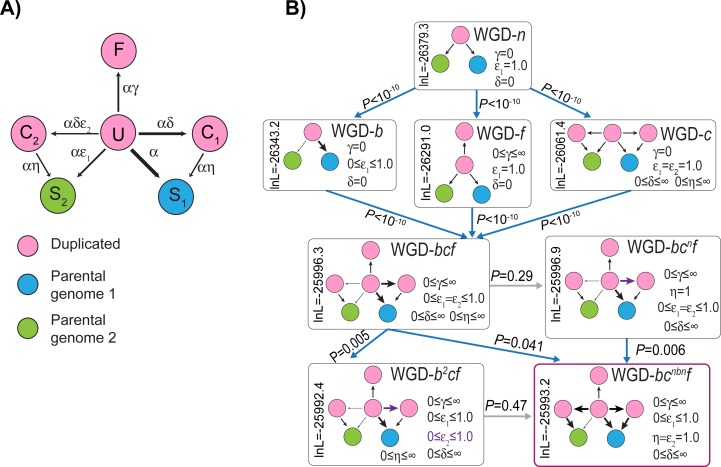
Testing nested models of post-WGD ohnolog evolution. **A)** Model states and parameter definitions for the set of models considered. **U** (Unduplicated), **C**_**1**_ (Converging state 1), **C**_**2**_ (Converging state 2) and **F** (Fixed) are duplicated states, while **S**_**1**_ (Single-copy 1) and **S**_**2**_ (Single-copy 2) are single-copy states (see Methods). **C**_**1**_ and **S**_**1**_ are states where the gene from the less-fractionated parental subgenome will be or are preserved, and **C**_**2**_ and **S**_**2**_ the corresponding states for the more-fractionated parental subgenome. The fractionation rate ε (the probability of the loss of a gene from the less fractionated subgenome relative to the more fractionated one) can either be the same for conversions to **C**_**1**_ and **C**_**2**_ as it is for **S**_**1**_ and **S**_**2**_ (ε_1_ = ε_2_) or it can differ (see **B**). The weights of the various arrows give a cartoon impression of the relative frequency of the different events: exact parameter estimates for the WGD-*bc*^*nbn*^*f* model are given in Fig 1. **B)** Testing nested models of WGD resolution. The most basic model (top) has neither biased fractionation nor duplicate fixation nor convergent losses. Adding any of these three processes improves the model fit (second row; blue arrows indicating statistical significance; *P*<10^−10^). Adding the remaining two processes also improves the fit in all three cases (WGD-*bcf* model in the third row; *P*<10^−10^). However, there is no evidence that the ε_2_ parameter is significantly different from 1.0 (WGD-*b*^*2*^*cf* does not improve the fit over WGD-*bc*^*nbn*^*f*, gray arrow indicating a lack of significant improvement in fit from the more complex model), implying no biased fractionation in the transitions to states **C**_**1**_ and **C**_**2**_. Likewise, there is no evidence that the η parameter is significantly different from 1.0 (WGD-*bcf* does not improve fit over WGD-*bc*^*n*^*f*), meaning that losses from **C**_**1**_ and **C**_**2**_ occur at similar rates as do losses from **U**. Hence, the WGD-*bc*^*nbn*^*f* model is best supported by these data and is used for the remaining analyses. Model names: WGD-*n*: Null model; WGD-*b*: Biased fractionation model; WGD-*f*: Fixation model; WGD-*c*: Convergence model; WGD-*bcf*: Bias/Convergence/Fixation model; WGD-*bc*^*n*^*f*: Bias/ Convergence (non-biased)/Fixation model; WGD-*b*^*2*^*cf*: Bias (2 rate)/Convergence/Fixation model; WGD-*bc*^*nbn*^*f*: Bias/Convergence (non-biased convergence, neutral convergent loss)/ Fixation model.

I analyzed the pillars with POInT [61], which uses the copy-number status of each pillar in each genome, which is either duplicated (states U, F and C_1_/C_2_ in Fig 2) or single-copy (states S_1_ and S_2_), as states in a phylogenetic model, allowing me to track resolution of the TGD along a tree in a manner similar to how DNA sequence evolution is modeled [64]. POInT’s evolutionary models include as parameters both the phylogeny of the species considered as well the orthology relations of the extant and lost genes in their genomes. Unlike all other model-based approaches to gene family evolution, POInT uses synteny data to condition the orthology estimates at each pillar on those at the neighboring pillars.

Because POInT infers orthologous chromosome segments based on a common gene order and shared gene losses, it requires an estimate of the order of the pillars in the ancestral genome immediately prior to the TGD [e.g., as was previously done for yeast; 65]. The TGD is considerably older and the genomes involved more rearranged than was the case for the polyploidies we previously analyzed [58, 61, 66]. Hence, I explored several means for estimating this order (*Methods*): different potential orders were compared based on the number of synteny breaks they induced. While the number of such breaks in the orders estimated for the TGD was larger in proportion to the number of pillars than was the case for our previous work, among the nearly optimal orders, POInT’s estimates of the model parameters are quite consistent (S1 Table). Hence, for the remainder of the analyses, I used the ancestral order with the highest ln-likelihood under the WGD-*bc*^*nbn*^*f* model (Fig 2). Similarly, the use of stringent homology criteria (see Methods and S2 Table) and the requirements for synteny yield a set of DCS blocks that represent a conservative set of loci with which to study the resolution of the TGD (*Methods*).

### Ohnolog fixation, biased fractionation and convergent losses are all observed after the TGD

A pair of homologous genes from different teleost genomes that survive from the TGD may either be orthologs or paralogs. POInT resolves this ambiguity by computing the likelihood of all *2*^*n*^ possible orthology states at each pillar (where *n* is the number of genomes), conditioned on the pillars to the right and left. We can the visualize the history of regions of these genomes by selecting the orthology relationship with highest posterior probability (Fig 1). Note that this orthology inference procedure accounts for the reciprocal gene losses that can create single-copy paralogs in taxa sharing a WGD [36]; it is distinct from the generic orthology inference approaches used for non-polyploids [67, 68].

I fit nested models of WGD evolution (Fig 2) to the DCS blocks in order to assess which of three processes observed after other WGD events were also detected after the TGD. The first process is duplicate fixation, meaning that some ohnolog pairs persist across the phylogeny longer than would be expected. The second process is biased fractionation, meaning that ohnolog losses favor one of the two parental subgenomes (“Less fractionated parental subgenome” in Fig 1), and the third is the presence of convergent losses. These losses represent overly frequent parallel losses of the same member of the ohnolog pair on independent branches of the phylogeny. No matter what the order that these three phenomena are added to the duplicate loss model, all three are independently statistically significant (*P* <10^−10^; Fig 2).

In models without biased fractionation (WGD-*n*, WGD-*f* and WGD-*c* in Fig 2), genes are assigned to each subgenome with equal probability. When biased fractionation is added (e.g., ε<1.0), those probabilities are allowed to differ, meaning that there can be a more fractionated subgenome with fewer surviving genes and a less fractionated one retaining more genes. Because it is reasonable to assume that autopolyploidies do not resolve themselves through biased fractionation, the presence of such bias is an indirect indicator of allopolyploidy [4]. It is important to note that POInT’s inferences regarding the presence of biased fractionation are conditioned on this uncertainty in subgenome assignment. One might think that the stochastic patterns of gene loss in DCS blocks would invariably cause POInT’s models to infer the presence of biased fractionation. However, we have previously shown that such is not the case: the yeast WGD event does not show significant evidence for a global pattern of biased fractionation despite being a known allopolyploid [58, 69]. As shown in S1 Fig, the pattern of shared losses allows the assignment of genes to “local” subgenomes with high confidence even without including biased fractionation in the model (ε = 1.0, WGD-*fc* model including convergent losses and duplicate fixation). Adding biased fractionation to the model allows local regions of the ancestral order to be globally phased into a more and a less fractionated subgenome. In S2 Fig, I show a set of inferred blocks where 7,6,5 or 4 of the teleost genomes agree from pillar to pillar in their identification of each subgenomes at a confidence of 80% both with and without the assumption of biased fractionation. For the 8 blocks that are larger than 100 pillars, I also separately fit the WGD-*f* and WGD-*bf* models and computed the significance of the observed pattern of fractionation (S2 Fig). Clearly, although the strength of the bias varies, it is a genome-wide pattern. We have previously argued that it is parsimonious to argue that all genes from the less fractionated blocks derive from a single parental subgenome [58], but this hypothesis is not a formal feature of the model.

**Fig 3:**
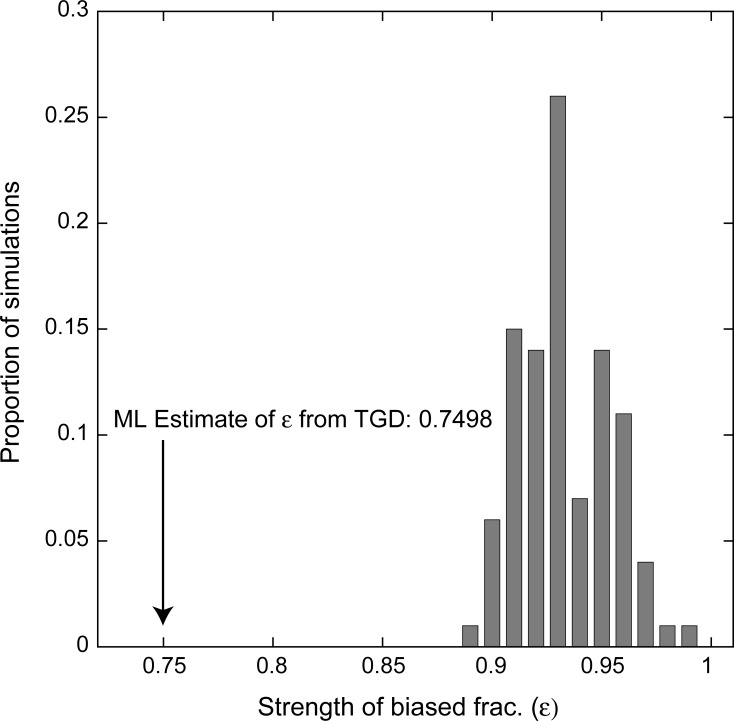
The estimated value of the biased fractionation parameter ε in the real teleost genomes (WGD*-bf* model, arrow, see Methods) is significantly different than those estimated from simulated genomes where biased fractionation was explicitly not included in the model (e.g., simulated ε = 1.0, bars). Estimates of ε from these 100 simulations are always less than 1.0 because the model fits stochastic variations in the preservation patterns as potential biased fractionation. However, this stochastic variation never yields estimates of ε as small as seen in the real dataset (*P*<0.01).

As mentioned, one might still argue that, because each synteny block will have some variation in loss patterns, the inference of presence of biased fractionation itself is only an artifact of stochastic variation in the blocks’ loss patterns. To firmly refute this possibility, I applied a new simulation-based statistical test for biased fractionation. First, I simulated sets of 8 genomes with POInT under a model without biased fractionation (WGD-*f*): these simulated genomes maintain the synteny blocks from the original genomes but have balanced gene losses within them. For each simulation, I then estimated the value of the ε parameter under a model with a bias (WGD-*bf*, see Methods), allowing me to assess what degree of spurious bias might be induced by our approach. The level of biased fractionation seen after the TGD is inconsistent with purely stochastic variation (*P<*0.01, Fig 3), strongly supporting the conclusion that biased fractionation occurred after the TGD.

### Inferring sets of retained and lost ohnologs from the TGD

From the WGD-*bc*^*nbn*^*f* model, I obtained lists of surviving ohnolog pairs from zebrafish (*Dr_Ohno_all* and *Dr_Ohno_POInT*, for all zebrafish ohnologs and zebrafish ohnologs also found syntenically in other genomes, respectively; see Methods*)*, the corresponding single-copy gene sets (*Dr_Sing_all* or *Dr_Sing_POInT*) and a set of early and late ohnolog losses (e.g., losses along the root and zebrafish tip branches of Fig 1A: *POInT_RootLosses* and *POInT_DrLosses*, respectively, see Methods). These gene sets allowed me to explore the associations between gene function and ohnolog survival post-TGD.

### Ohnolog pairs are unusually rare amongst genes expressed in the earliest stages of development

**Fig 4:**
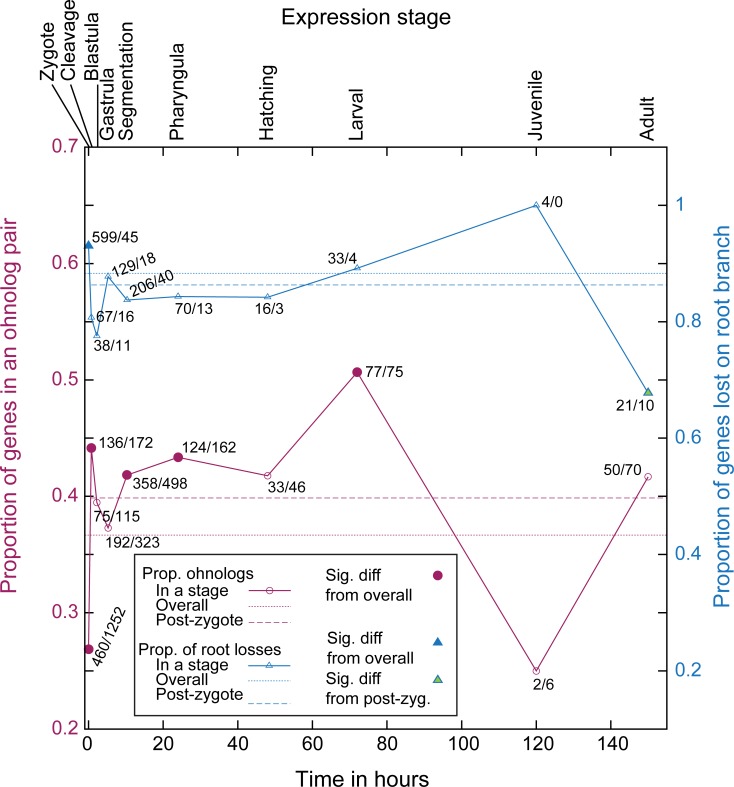
Timing of gene expression in development compared to patterns of ohnolog loss and retention. On the *x*-axis is a timeline of zebrafish development from ZFIN [72], with the relevant stage names indicated at the top. The trendline in red indicates the proportion of zebrafish genes with an ohnolog partner *first* expressed at that stage (relative to total number of zebrafish genes analyzed with POInT and expressed at that stage). The dotted red line is the overall proportion of genes with an ohnolog partner in the POInT dataset (Dr_Ohno_POInT), while the dashed line is this proportion excluding any genes expressed in the zygote (see Methods). Open points show no statistically distinguishable difference from the overall proportion [chi-square test with an FDR correction, P>0.05; 74]. Red-filled points are significantly different from this overall mean (*P≤*0.05). Each point is labeled with the number of genes first expressed at that stage that have a surviving ohnolog and the number that do not. Trendlines in blue show similar values comparing the set of genes that POInT predicts were returned to single copy along the root branch of Fig 1 (confidence ≥ 0.85) to those only returned to single-copy along the tip branch leading to zebrafish. Hence, the right *y*-axis gives the proportion of losses that occurred along the root branch (relative to the sum of that number and the number of losses along the zebrafish branch). The dotted blue line is the overall proportion of genes returned to single-copy on the root branch (scaled as just described) while the dashed line is this proportion excluding any genes expressed in the zygote (see Methods). Open points are not statistically different from the overall proportion [chi-square test with an FDR correction, P>0.05; 74]. Blue-filled points are significantly different from this mean (*P≤*0.05), while green filled points are also different from the mean seen when zygotic-expressed genes are excluded (*P≤*0.05). Each point is labeled with the number of genes first expressed at that stage that returned to single copy along the root branch and along the branch leading to zebrafish.

As mentioned, the TGD affords the opportunity to study the effects of WGD on developmental evolution. We had speculated that genes used in the earliest stages of development might be overly likely to be preserved in duplicate after WGD because the noise buffering effects of gene duplication might be beneficial at such times [70, 71]. However, such is not the case: genes with mRNAs present in the zygote were much *less* likely to be preserved as ohnolog pairs than genes *first* expressed later in development [Dr_Ohno_POInT versus Dr_Sing_POInT, data from the ZFIN database, Fig 4; 72]. I wondered if this observation might be driven by a dearth of ohnolog pairs among those genes where mRNA transcribed from the maternal genome is used in the early embryo (maternal mRNAs), since such parent-offspring transmission might be disrupted in an allopolyploidy. To test this idea, I used data from Aanes et al., [73], who have partitioned the mRNAs present in the earliest stages of zebrafish development into three groups: maternal transcripts and those seen prior to and after the mid-blastula transition. As Table 1 shows, there is some deficit of ohnologs amongst the maternally-expressed genes, but its significance depends on the ohnolog set used, and there is no excess of early duplicate losses among this set. In contrast, the genes expressed from the embryonic genome prior to the MBT are strongly depleted in ohnologs and the single-copy genes in question are more likely to have returned to single copy along the root branch than expected (Table 1). There is then relatively little signal of ohnolog excess or deficit amongst the genes expressed later in development (post-MTB).

**Table 1:** Expression timing and fate of TGD-produced ohnologs.

Expression cluster	1^st^ gene set	2^nd^ gene set	Prop. of 1^st^ set in cluster^a^	Prop. of 2^nd^ set in cluster^a^	*P*^b^
Maternal transcripts^c^	*Dr_Ohno_all*^d^	*Dr_Sing_all*^d^	0.03 (116/4279)	0.04 (484/11616)	**2.4x10**^**-5**^
	*Dr_Ohno_POInT*^e^	*Dr_Sing_POInT*^e^	0.03 (81/2552)	0.04 (193/4408)	**0.015**
	*POInT_RootLosses*^f^	*POInT_DrLosses*^f^	0.05 (103/1894)	0.03 (8/250)	0.18
Pre-MBT transcripts^g^	*Dr_Ohno_all*	*Dr_Sing_all*	0.10 (435/4279)	0.15 (1709/11616)	**1.2x10**^**-13**^
	*Dr_Ohno_POInT*	*Dr_Sing_POInT*	0.11 (284/2552)	0.17 (762/4408)	**3.9x10**^**-12**^
	*POInT_RootLosses*	*POInT_DrLosses*	0.19 (351/1894)	0.12 (31/250)	**0.022**
Zygotic transcripts^h^	*Dr_Ohno_all*	*Dr_Sing_all*	0.06 (250/4279)	0.05 (573/11616)	**0.024**
	*Dr_Ohno_POInT*	*Dr_Sing_POInT*	0.06 (142/2552)	0.05 (216/4408)	0.25
	*POInT_RootLosses*	*POInT_DrLosses*	0.05 (92/1894)	0.05 (12/250)	>0.95

**a:** Proportion of all genes in the set (see left) that were observed to be expressed in the cluster in question, with the total number of expressed genes over the total number of genes in that set given in parentheses.

**b**: *P-*value for the hypothesis test of equal proportion of genes in both sets falling into the expression cluster (chi-square test with 1 degree of freedom)

**c:** Genes determined by Aanes et al., [73] to have been expressed in the developing embryo from maternally-derived transcripts.

**d**: Comparison of all identified zebrafish ohnologs to all zebrafish single-copy (with respect to the TGD) genes, comprising 15,895 of the 19,436 zebrafish genes with gar homologs. See Methods for further details.

**e**: Comparison of all zebrafish ohnolog pairs found in the 8-species POInT analysis to the corresponding zebrafish single-copy (with respect to the TGD) genes. See Methods for further details.

**f**: Comparison of zebrafish single-copy genes inferred to have been lost on the common root branch of Fig 1 to zebrafish single-copy genes inferred by POInT to have been lost after the zebrafish/cavefish split (inference confidence ≥ 0.85 in both cases). See Methods for further details.

**g:** Genes determined by Aanes et al., [73] to have been expressed in the developing embryo prior to the mid-blastula transition (<3.5 hours post-fertilization).

**h:** Genes determined by Aanes et al., [73] to have been expressed in the developing embryo only after the mid-blastula transition (>3.5 hours post-fertilization).

### GO analyses show similar patterns of ohnolog loss and retention as seen for other ancient polyploids

I used the PANTHER classification system [75] to look for over and under-represented functions among the surviving ohnologs (and among the early ohnolog losses) in zebrafish. S3 Table gives the complete list of significantly over and under-represented GO terms across the three hierarchies (molecular function, biological process, and cellular compartment). Here I discuss some notable results from the *Dr_Ohno_POInT* to **Dr_Sing_POInT comparison**

Some of the Molecular Function terms over-represented among surviving ohnologs mirror results from other polyploids, such as “kinase activity” (*P =* 0.008) and “sequence-specific DNA binding transcription factor activity,” (*P =* 0.02). Many of the Biological Process terms found to be over-represented involve aspects of nervous system function: “nervous system development” (*P<*10^−5^), “neuron-neuron synaptic transmission” (*P<*10^−5^), “synaptic vesicle exocytosis” (*P =* 0.0014) and “sensory perception” (*P<*10^−4^), a pattern consistent with previous analyses of the role of the surviving ohnologs from the TGD [76].

I was particularly interested to see if the terms associated with fewer than the expected number of ohnologs might shed any light on the relative absence of ohnologs among the mRNAs present in the earliest stages of development. And indeed, the four most statistically under-represented Biological Process terms among the surviving ohnologs (excepting “Unclassified”) were “DNA metabolic process,” “translation,” “tRNA metabolic process” and “RNA metabolic process” (*P<*10^−4^ for all). The four most significantly under-represented Molecular function terms (again excepting “Unclassified”) were “methyltransferase activity,” “structural constituent of ribosome,” “nuclease activity” and “nucleotidyltransferase activity” *(P<*10^−3^ for all). Since the earliest cell divisions in the embryo do not involve cell-type differentiation, the over-abundance of single-copy genes with roles in basic cellular processes (which would be needed even prior to such differentiation) is in accord with the expression timing results above.

I also considered a set of 132 ohnolog pairs preserved across all eight species *(POInT AllOhnologs; Methods*): in this case the patterns of ohnolog over- or under-representation across functions are different. Few molecular function terms are over-represented, while biological processes and cellular compartments related to neuron development are over-represented among genes with surviving ohnologs in all eight species (S3 Table). I speculate that while selection to maintain relative dosage (e.g., the DBH) results in the retention of certain gene classes, those dosage constraints can be later relaxed [49] in independent lineages (for instance through new gene regulatory circuits), meaning that the duplicates preserved in this manner in modern genomes will differ across those lineages. This proposal would explain why the DBH-consistent patterns seen among zebrafish ohnologs are not seen for this shared set.

### Ohnolog pairs are unusually abundant in certain nervous and sensory tissues

Using ZFIN data [72] on the anatomical locations of gene expression, I asked whether any embryological tissues had more or fewer members of ohnolog pairs expressed in them than expected, given the number of single-copy genes active in these same locations. Relative to the corresponding single copy genes (*Dr_Sing_POInT*), ohnologs (*Dr_Ohno_POInT*) are excessively likely to be expressed in the brain, diencephalon and epiphysis of the segmentation stage, (10.33–24 hours) and in the olfactory epithelium, retinal ganglion cell layer, and the retinal inner nuclear layer of the pharyngula stage [24–48 hours, P<0.05, chi-square test with FDR multiple test correction; 74]. All of these locations except the olfactory epithelium also showed a significant excess of expressed ohnologs relative to single copy genes when the full set of zebrafish ohnologs was used (*Dr_Ohno_all* versus *Dr_Sing_all*, S4 Table). When I considered ohnologs preserved across all eight genomes (*POInT AllOhnologs* versus *POInT AllSingle)*, there were no tissues significantly enriched in ohnologs, likely due to the small number of such universally conserved duplicates (S4 Table).

One concern with this analysis might be that the data in ZFIN are biased toward surviving ohnologs: however this does not appear to be the case: 58% of ohnologs (*Dr_Ohno_POInT*) were identified in at least one anatomical location, which is actually less than the 61% of the single-copy genes (*Dr_Sing_POInT*) so identified.

### The TGD and the organization of the teleost retina

The overrepresentation of ohnologs in genes expressed in parts of the retina was intriguing because teleost fishes organize the photoreceptor cells in their retinas into a regular mosaic with defined positions for the cone cells with differing wavelength sensitivities [77–79]. This organization is not ancestral to vertebrates, and there is evidence that it might be an innovation due to the TGD: spotted gar lacks both this trait and the TGD [14, 80]. I conducted a GO analysis of all ohnologs and single-copy genes expressed in either the ganglion or inner cell layers of the retina at the pharyngula stage of development. No terms associated with biological process were over-represented in either tissue, and no terms associated with molecular function were over-represented in the inner cell layer. However, for the ganglion layer, the term “transmembrane transporter activity” was significantly overabundant among the surviving ohnologs (*P* = 0.044 after FDR correction). Moreover, while the retinal inner nuclear layer does not show an excess of surviving ohnologs preserved in all eight teleost genomes (*P =* 0.10), it *does* show such an excess when ohnologs preserved in every genome *except* that of the cavefish [which was derived from cave-dwelling individuals with reduced eyes; 81] are considered (*P =* 0.041). Likewise, the expression of duplicated genes from the TGD in these locations are probably not specific to zebrafish. The only two GO biological process terms that are globally *under* represented among the genes returned to single-copy along the root branch of Fig 1 (e.g., terms that are characteristic of genes that survived in duplicate at least to the first post-TGD speciation) are “synaptic transmission” and “cell-cell signaling.” The single Cellular Compartment term similarly under represented is “neuron projection” (S3 Table). These annotations, while not specific to retinal development, may nonetheless be suggestive. Genes returned to single copy along the root branch are also less likely than expected to be expressed in the retinal ganglion cell layer (*P =* 0.02). Collectively, these results suggest that the duplicated genes created by the TGD were likely involved in subsequent evolution changes in neuronal development, accounting for their retention as ohnologs across the teleost phylogeny (e.g., including ohnologs retained in all eight species; see S3 Table).

### Surviving TGD ohnologs are less likely to be essential

I compared the proportion of phenotyped genes with surviving ohnologs judged to be essential in zebrafish to the same proportion among those genes without surviving ohnologs: the genes with ohnolog partners are less likely to be essential (Table 2, see Methods). Importantly, this effect is not a result of any intrinsic feature of these genes: when examining the two groups in the unduplicated outgroup mouse, I find that that single-copy mouse orthologs of the duplicated and the unduplicated zebrafish genes have similar essentialities in that animal. However, I also note that this effect is not a strong one: when I examined the smaller set of ohnologs with support across the eight genomes (*Dr_Ohno_POInT* versus *Dr_Sing_POInT*), the proportions shown in Table 2 are nearly identical, but the effect is non-significant due to the smaller sample size (*P =* 0.14, chi-square test).

**Table 2:** Essentiality and the TGD.

Essentiality data	Prop. of phenotyped genes with an ohnolog that are essential	Prop. of phenotyped genes without an ohnolog that are essential	*P*^a^
Zebrafish^b^	0.062 (6/97) ^c^	0.145 (46/318) ^d^	**0.048**
Mouse^e^	0.556 (42/72) ^f^	0.506 (161/318) ^g^	0.53

**a:** *P-*value for the hypothesis test of equal proportion of essential genes in *Dr_Ohno_all* vs *Dr_Sing_all*.

**b**: Essentiality defined as genes in the ZFIN database [72] phenotyped as “lethal,” “dead” or “inviable.”

**c**: Numbers in the parenthesis give the number of essential genes over the total number of ohnologs in the set (*Dr_Ohno_all*).

**d**: Numbers in the parenthesis give the number of essential genes over the total number of single-copy genes in the set (*Dr_Sing_all)*.

**e**: Essentiality defined by the International Mouse Phenotyping Consortium’s list of essential mouse genes [82, 83].

**f**: Numbers in the parenthesis give the number of essential genes over the total number of ohnologs in the set (*Dr_Ohno_all*). Note that ohnolog pairs in zebrafish are by definition single-copy in gar and mouse, accounting for the smaller number of comparisons.

**g**: Numbers in the parenthesis give the number of essential genes over the total number of single-copy genes in the set (*Dr_Sing_all*).

### TGD ohnologs lie in connected parts of the zebrafish metabolic network

I examined the position of the ohnolog pairs in the published zebrafish metabolic network [84]. In this network, enzyme-coding genes are nodes and pairs of nodes are connected by edges if their corresponding reactions share a metabolite (*Methods*). Ohnologs are more likely to be members of this network than are single copy genes (*P =* 0.0005 and *P =* 0.025 for *Dr_Ohno_all* versus *Dr_Sing_all* and *Dr_Ohno_POInT* verse *Dr_Sing_POInT*, respectively). Ohnolog pairs also occupy more connected parts of this network (e.g., they share metabolites with more other reactions; Table 3). The ohnologs do not differ from single copy genes in their clustering coefficients [the propensity of connected nodes to have common neighbors; 85] or betweenness-centrality [the number of the network’s shortest paths passing through a given node; 86].

**Table 3:** The TGD and the zebrafish metabolic network.

Network statistic	Ohnolog datasets compared	Mean ohnolog value^a^	Mean single-copy gene value^b^	*P*^c^
Node degree^d^	*Dr_Ohno_all/Dr_Sing_all*^e^	30.9	21.4	**0.002**
	*Dr_Ohno_POInT Dr_Sing_POInT*^f^	31.5	23.2	**0.032**
Avg. clustering coeff.^g^	*Dr_Ohno_all/Dr_Sing_all*^e^	0.78	0.77	>0.5
	*Dr_Ohno_POInT Dr_Sing_POInT*^f^	0.77	0.76	>0.5
Mean # shortest paths^h^	*Dr_Ohno_all/Dr_Sing_all*^e^	18020	12904	0.07
	*Dr_Ohno_POInT Dr_Sing_POInT*^f^	19280	14132	0.18

**a**: Mean value of the statistic in question for the ohnolog pairs (ohnolog pairs were merged and averaged prior to computing the global average).

**b:** Mean value of the statistic in question for the single-copy genes.

**c**: *P-*value for the hypothesis test of equal mean statistic value for the ohnologs and single-copy genes (Network randomization test; *Methods*).

**d**: Number of edges per network node.

**e**: Comparison of all identified zebrafish ohnologs to all zebrafish single-copy (with respect to the TGD) genes. See Methods for further details.

**f**: Comparison of all zebrafish ohnolog pairs used in the 8 species POInT analysis to the corresponding zebrafish single-copy (with respect to the TGD) genes. See Methods for further details.

**g**: Ratio of the number of edges between each triplet of nodes to the maximum number of such connections possible [85].

**h**: The mean of the number of shortest paths through the network that pass through a given node, also known as betweenness-centrality [86].

## Discussion

Polyploidies of differing ages are ubiquitous across the tree of life [6], yet many of the studies of polyploidy’s genome-wide effects have focused on relatively recent events. Thus, while we know quite a bit about the fate of individual ohnolog pairs surviving from the TGD and the vertebrate 2R events [11, 28–30, 32, 33, 87, 88], we do not know whether the patterns of genome evolution, such as adherence to the DBH and the occurrence of biased fractionation, seen after more recent polyploidies, also apply to these ancient ones. Existing data should also be interpreted with some caution, as the methods used to identify the relics of ancient WGDs are subject to bias. Hence, Inoue et al.,’s estimates [39] of the timing of ohnolog losses after the TGD differ from those presented here, with their estimates of the proportion of losses along the root branch (which in both analyses ends with the split of cave fish and zebrafish from the other taxa studied) being >1.5 greater than that estimated with POInT, with an average of only 21% as many proportional losses inferred along the tip branches as POInT predicts. The reason for the discrepancy is likely that Inoue et al.,’s gene tree-based method cannot invariably phase post-WGD orthologs. Without such phasing, independent losses in different lineages will be mistaken for shared losses, leading to the over-estimates of initial loss rates.

The data shown here support a role for the DBH in resolving the TGD: the location of ohnologs in the zebrafish metabolic network is similar to the pattern seen in the network of the polyploid plant *Arabidopsis thaliana* [89] and the classes of ohnologs retained follow the predictions of the DBH [45, 49]. However, further work will be needed to assess whether these surviving ohnologs with high interaction degree are still be maintained by selection on relative dosage or if some other force is now at work [49]. Of course, any deep-time comparative genomics study also suffers from the caveat that the genes in each species for which homology is unclear may differ in their evolutionary patterns from those compared across the genomes. In the case of this study, any ohnolog pairs that have undergone rearrangement in all eight species, as well as other fast-evolving genes, will not have been included in our POInT analyses and may display other modes of evolution.

The TGD also appears to have been an allopolyploidy, as had been speculated by Christensen and Davidson [90], because there is strong evidence for biased fractionation. While Makino and McLysaght [91] have shown that physical interactions between neighboring genes can produce *local* biases in post-WGD loss patterns, this mechanism appears unlikely to generate the genome-wide preference for a single parental subgenome that was seen with the TGD. And indeed the biases seen by Makino and McLysaght could, as they note, be due to allopolyploidy. The pattern of biased fractionation seen after the TGD is also consistent with that seen after polyploidies in plants [4, 58, 69].

The association between when genes are expressed in development and their evolutionary response to the TGD is also of interest. It was already known that surviving ohnolog pairs in zebrafish were unlikely to be expressed in the earliest phases of development [92], a pattern attributed to preferential retention of such pairs from genes expressed later in development. Here, I have shown that this dearth of ohnologs among the zygotically-expressed genes was a pattern driven by gene loss events in the early evolutionary history of the TGD, prior to the first speciation between the eight species studied. Viewed in this light, association of expression timing and preservation recalls patterns seen in plants and yeast, where processes such as DNA repair were rapidly returned to single copy after polyploidy [66, 93]. Indeed, “DNA repair” is a highly under-represented term (*P<*10^−3^) among the zebrafish TGD ohnologs, though not one of the top four listed above. De Smet et al., have argued that these loss patterns suggest selection to return genes with these types of function to single copy. Hence, another explanation for the lack of zygotically-expressed ohnolog pairs could be selection against maintaining them in duplicate in the early phases of the resolution of the TGD. In this view, the causality in the association is driven by the molecular functions, such as DNA repair, and the observation that losses are more common in early-expressed genes merely reflects the fact that such functions are over-represented in genes expressed in these stages. Moreover, this lack of early-expressed ohnologs arithmetically corresponds to an excess of them involved in other processes such as multicellular development. Hence, polyploidy in multicellular organisms might concentrate its effects in such developmental processes [94].

In this vein, the apparent over-abundance of ohnologs expressed in the developing retina, a pattern also recently observed by Parey et al., [95], is interesting because work in the spotted gar strongly suggests that the mosaic organization of the photoreceptor cells in teleost retainae [77–79] represents a morphological innovation whose evolutionary appearance was coincident with the TGD [80]. Not only are ohnologs over-represented in genes expressed in some of the retinal layers, but a GO analysis suggested that many of these duplicated genes function as transmembrane transporters. Several analyses have suggested that cell-to-cell communication in the early stages of retinal development may drive the mosaic organization [79, 96], and such transmembrane proteins are obvious candidates for such communication.

The more general pattern of over-retention of duplicate genes functioning in the nervous system has been previously reported with both with respect to the TGD [97] and for other vertebrate WGDs [76, 98, 99]. Roux, Liu and Robinson-Rechavi argue that purifying selection opposing the appearance of sequence variants of duplicate genes expressed in neural tissues has the indirect effect of preventing the loss of the duplicates themselves [76]. This argument also links to another proposed explanation of the convergent patterns of ohnolog loss and preservation across divergent taxa: the hypothesis that genes that tend to experience autosomal dominant mutations may be overly likely to survive in duplicate due to the selective sweeps that clear these dominant mutations from the population after polyploidy [100]. This hypothesis requires further research, both because the degree to which it is distinct from the dosage balance hypothesis (where genes likely to show dominant mutations may also be likely to be dosage sensitive, if both phenotypes are driven by the appearance of aberrant interactions with other gene products) and because De Smet et al., [93] have suggested that selection to *remove* genes subject to such dominant lethal mutations is behind the rapid deletions of DNA repair enzymes after polyploidy.

The evolution of gene expression after the TGD more generally has also been studied: perhaps the most interesting resulting observation was that pairs of ohnologs taken together show greater expression similarity to their single-copy gar orthologs than do the two genes considered individually [101]. It is tempting to go further and to attempt to infer genes that have undergone sub- or neofunctionalization in their expression patterns post-TGD. However, as we have pointed out in the past [49], the potential for neutral drift in expression levels makes such analyses prone to false positives unless the underlying expression data are deeply sampled and analyzed phylogenetically with Ornstein-Uhlenbeck-type models of continuous character change [102].

While duplicate genes can provide a “backup” for each other in response to gene knockout, this effect is expected to degrade as the pair ages [103], making the apparent rarity of essential genes among the ancient ohnolog pairs of the TGD a bit surprising. However, essentiality and duplication interact in complex ways. On the one hand, a gene’s propensity to duplicate is associated with whether or not it is essential: small scale duplications favor less essential genes [104], but post-WGD evolution appears to neither favor nor disfavor the retention of (formerly) essential genes after WGD [105, 106]. Gene duplication then apparently imparts the partial redundancy seen in studies of yeast, nematodes and mice [103, 107, 108]. I suspect that the combined observation of reduced essentiality among zebrafish ohnologs with no reduction in the essentiality of their single-copy mouse orthologs mostly likely represents surviving shared functions between ohnolog pairs that were preserved in duplicate due to other selective pressures.

The most general message apparent from these analyses is that polyploidy shapes the evolutionary trajectories of its possessors over very long time scales, both through first-order effects such as genetic robustness, and, more importantly, through the appearance of duplication-driven evolutionary innovations. Examples such as the changes in retinal structure described are particularly important because they are a class of innovations requiring changes in many genes at once, meaning that they may have only been feasible with the large number of duplicates induced by polyploidy. Though relatively few examples of such innovations are currently known [109–112], as our knowledge of both polyploidy and the systems biology of the cell increases, it is likely more will be found.

## Methods

### Identifying the relics of the TGD from double-conserved synteny blocks

I applied our pipeline for inferring shared blocks of DCS [58] to eight polyploid fish genomes, taken from Ensembl release 84 [113]: *Astyanax mexicanus* [Cave fish; 81], *Danio rerio* [Zebrafish; 114], *Takifugu rubripes* [Fugu; 26], *Oryzias latipes* [Medaka; 115] *Xiphophorus maculates* [Platyfish; 116], *Gasterosteus aculeatus* [Stickleback; 117], *Tetraodon nigroviridis* [27] and *Oreochromis niloticus* [Tilapia; 118]. The genome of *Lepisosteus oculatus* [spotted gar; 101] was used as the unduplicated outgroup.

The pipeline has three steps. First, I performed a homology search of each polyploid genome against that of gar with GenomeHistory [119]. I defined a gene from a polyploid genome and a gar gene to be homologs if they had a BLAST E-value [120] ≤10^−8^ and were >60% identical at the amino acid level. I further required that the length of the genes’ pairwise alignment be 65% or more of their mean length and that the pair have nonsynonymous divergence (K_a_) less than 0.6. These parameters give good coverage of the genomes involved: between 70% and 80% of gar genes have a homolog in each genome with the TGD, and 70% to 82% of genes in those genomes have a gar homolog. Nonetheless, the parameters do not overly merge gene families: 58% to 60% of the gar genes have only a single homolog in the TGD-possessing genomes.

This set of homologs was then the input to the second step of the pipeline: the inference of DCS blocks in each polyploid genome. This step determines which of the potentially many homologs of a given gene in gar are the ohnologs from the TGD. It does so by maximizing the number of homologs placed in the DCS blocks. The resulting set of these *n* pillars is denoted *A*_*1*_..*A*_*n*_. Each pillar has associated with it a set of homologous genes from the polyploid genome *h*_*1*_*…h*_*h*_. At most two of these homologs can be assigned to the pillar’s ohnolog positions, denoted *A*_*i*_*(p*_*1*_*)* and *A*_*i*_*(p*_*2*_*)*. We define *A*_*O(i)*_ to be the *i*^*th*^ pillar in the reordered version of this dataset. It is necessary to estimate the *A*_*O(i)*_s because the teleost genomes have undergone rearrangements since the TGD [121]. Using simulated annealing [122, 123], I sought the combination of homolog assignments and pillar order that maximizes the number of pillars where the genes in neighboring pillars are also neighbors in their genome [58]. Precisely, I maximized the score *s* of such a combination of homolog assignments and pillar orders:

$$s=\sum_{i=1}^{n}\sum_{k=1}^{2}\begin{array}{c} 1\\ 0 \end{array}|\begin{array}{l} A_{O(i)}(p_{k})\;and\;A_{O(i+j)}(p_{k})\;are\;neighbors\\ \;otherwise \end{array}$$ (1)

Here *j* represents the number of pillars to the right one must move before finding the next gene in that track (*j≥1*). Once those inferences were complete for each of the eight polyploid genomes, I merged them by using the gar genes as references. Taking a conservative approach, I retained pillars only if each assigned homolog from every genome had at least one syntenic neighbor in the inferred order. The result was 5589 pillars with at least one syntenic gene from each polyploid genome. I then again used simulated annealing to infer the optimal pillar order over all eight genomes. Because of the high degree of rearrangement, I made inferences of the optimal ordering under three different criteria. First, I started with the order of the gar reference genes and sought orderings with the fewest total synteny breaks (*Naïve_Opt*) [58]. Second, I used an initial greedy search to place pillars with many neighboring genes in the eight extant teleost genomes near to each other, which reduced the number of initial breakpoints by about 30%. I then again sought an order with minimal breaks (*Greedy_Opt*). Finally, I sought an ordering that maximized the number of neighboring pillars having no synteny breaks between them in any genome and, after using this optimization criterion for several iterations, again applied the standard search for the fewest total breaks (*Global_Break_Opt*). I then used the inferred order that gave the highest likelihood of observing that WGD data under the WGD-*bc*^*nbn*^*f* gene loss model (S1 Table; see *Modeling the evolution of the TGD* below) for all further analyses.

I note that the *Naïve_Opt* and *Greedy_Opt* criteria have the undesirable tendency to favor orders that place breakpoints on the branch shared by zebrafish and cavefish, since the other six species share a more recent common ancestor. As such, orders with relatively fewer breaks can be constructed by assuming rearrangements that occurred in the ancestor of these six genomes after their split from the other two are actually ancestral (see S2 Fig) and forcing the reciprocal rearrangement on to the shared zebrafish/cavefish branch. Unfortunately, breakpoints are not themselves evolutionary events but result from genome transpositions and inversions. Moreover, there are no exact algorithms for mapping from breakpoints to these true evolutionary events [124]. As a result, the standard approach of using parsimony to correct for evolutionary relationships when computing breakpoint scores is flawed. To assess the seriousness of this problem, I repeated the ancestral pillar order inference considering *only* breaks in the genomes of *T*. *nigroviridis* and *D*. *rerio*, which are the genomes with the fewest breaks in the upper and lower clades of the tree in Fig 1, respectively. Because only one genome from each clade is considered, the bias in breakpoint position is not seen (S3C Fig). The order produced by this optimization technique is suboptimal relative to *Greedy_Opt*, but the inferred orthology estimates are nonetheless very similar, with 75% of the pillars agreeing in their orthology inferences with ≤15% difference in their inferred confidence (S3A and S3B Fig). Estimates of the model parameters for the WGD-*bc*^*nbn*^*f* model for this order are given in S1 Table.

### Quality of the inferred double-conserved synteny blocks

Given the ancient nature of the TGD, it is reasonable to ask if this DCS inference protocol is sufficient. However, the mapping between the genomes possessing the TGD and spotted gar is less difficult than might be expected, with 69–71% of the genes in the teleost genomes in our final dataset having only a single gar homolog (and where that gar gene matches at most 2 genes in the genome with the TGD; S2 Table). Although I required every analyzed gene to be in synteny in Step 2 of the pipeline, the estimate of a global ancestral order requires breaking some of these synteny blocks. But this problem is not serious: >94% of the genes across all the genomes with the TGD that I analyzed are in synteny blocks in the estimated ancestral order used, with the large majority in blocks of 5 or more genes (S2 Table). I provide the synteny relationships under the inferred order for the eight genomes as supplemental data.

I also explored how well gene trees inferred from individual ohnolog pairs recapitulate the data I obtained with synteny-based methods. Of the 132 pillars in the dataset where all eight species share ohnolog pairs, there are nine pillars where all of the 16 genes that are members of these ohnolog pairs show syntenic associations in both directions. Such positions represent the best-case scenario for gene tree-based methods: the presence of ohnolog pairs is unambiguous and there are no confounding gene losses. I extracted the (9x8x2 = 144) genes in question and made codon-preserving alignments of them with T-Coffee [125]. Using phyml [126], I inferred maximum likelihood trees from these alignments under the GTR model with 4 categories of substitution rates that followed an estimated discrete gamma distribution. For none of the nine pillars was the expected pair of mirrored species trees inferred (see Fig 1). In fact, of the 18 gene trees inferred (two per ohnolog pair), only 3 matched the assumed species tree, and no other topology was more frequent. This result is unsurprising: the relationships in question are characterized by long branches and may experience gene conversion post-WGD [41, 127–129]. Hence, a gene-tree based approach to the TGD requires reconciling such gene trees with a proposed species tree using a tool such as NOTUNG [130]. While this approach can be quite successful, it does not easily allow the testing of alterative phylogenetic hypotheses (S3 Fig) and can be misled by certain types of reciprocal gene loss [40].

### Modeling the evolution of the TGD

I analyzed the DCS blocks from these genomes using POInT [61, 66] under several models of post-WGD duplicate loss. These models have four to six states (Fig 2): **U** (undifferentiated duplicated genes), **F** (fixed duplicate genes), **S**_**1**_ and **S**_**2**_ (single copy states) and the converging states **C**_**1**_ and **C**_**2**_. These last two states model the potential for the independent parallel losses first seen in yeast [40, 61]. I used likelihood ratio tests to identify the combination of these factors best fitting the data [Fig 2; 131]. POInT’s optimal orthology inferences for all pillars (which includes the POInT ohnologs and single copy genes for zebrafish, e.g., *Dr_Ohno_POInT* and *Dr_Sing_POInT*), its input data files for these analyses, my estimates of the conditional probabilities of all ohnolog transitions along each branch (the underlying data for the gene loss estimates in Fig 1), the supplemental figures, the underlying data from the manuscript figures, and the lists of all zebrafish ohnologs and single copy genes (*Dr_Ohno_all* and *Dr_Sing_all*) are all available on figshare: https://doi.org/10.6084/m9.figshare.11317760.v5; the POInT source code is available from GitHub: https://github.com/gconant0/POInT.

### Simulating genome evolution under a model where no biased fractionation occurs

We have previously described using POInT to simulate genome duplications [61]. Briefly, I started from a set of completely duplicated pillars and the assumed gene order previously estimated. In locations where gene losses in one genome had generated a synteny break (e.g., after *caln1* in Fig 1), I extended the left contig to include the introduced duplicates. Then, using the maximum likelihood estimates of the model parameters and branch lengths under the WGD-*f* model, I generated a new set of post-WGD duplicate losses along the phylogeny of Fig 1. Finally, I applied the “Tracking flip prob.” parameter noted in Fig 1 to model POInT’s estimated errors in orthology inference, introducing new synteny breaks in the simulated genomes whenever a uniform random number was drawn with a value less than this parameter. I analyzed 100 such simulated sets of genomes with POInT under the WGD-*bf* model (e.g., biased fractionation and fixation allowed, but the δ parameter in Fig 2 set to 0) and extracted the value of ε, which is plotted in Fig 3. No simulated dataset had a value of ε as small as seen in the real dataset (*P*<0.01).

### The TGD and the teleost phylogeny

I used the phylogeny of Near et al., [132] as the assumed phylogeny of these eight species: four near topological neighbors of this tree all gave lower likelihoods of observing the genomic data than it did (S4 Fig).

### Zebrafish ohnolog and single-copy gene sets

Based on the inferences above, I defined two sets of zebrafish ohnologs and corresponding single copy genes. *Dr_Ohno_all* is the set of all ohnolog pairs that are part of DCS blocks found in the pairwise comparison of *D*. *rerio* to gar; *Dr_Sing_all* gives the corresponding WGD loci that have returned to single copy. *Dr_Ohno_POInT* corresponds to the set of ohnologs from zebrafish for which the pillar in question was also identified in the other seven polyploid teleost genomes, with *Dr_Sing_POInT* being the corresponding single copy set. These POInT ohnologs overlap reasonably well with the larger set of zebrafish ohnologs inferred by Singh and Isambert [133], where 66% of them are also present. However, the overlap is smaller (43%) with the ohnolog set inferred by Braasch et al., [101], due to the smaller size of that list. I also defined a pair of gene sets consisting of genes that POInT predicts with high confidence (*P≥*0.85) to have been returned to single copy on the shared root branch of the phylogeny in Fig 1 (*POInT_RootLosses*) and a corresponding set predicted with the same confidence to have been lost only on the branch leading to the extant *D*. *rerio* (e.g., after the split of zebrafish and cavefish; *POInT_DrLosses*). Finally, I considered ohnologs shared by all eight species (*POInT AllOhnologs*), comparing these genes to genes that are single-copy in all eight genomes (*POInT_AllSingle*).

### Gene expression timing and WGD

From the ZFIN database [72], I extracted the earliest developmental stage at which each zebrafish gene’s transcript has been observed and the corresponding time of expression (hours post-fertilization). I also extracted all non-adult anatomical locations at which each gene’s transcript had been detected. For each developmental stage and location, I used a chi-square test with a false-discovery rate correction [74] to test for differences in the proportion of ohnologs and non-ohnologs (*Dr_Ohno_all* vs *Dr_Sing_all* and *Dr_Ohno_POInT vs Dr_Sing_POInT*) expressed at that location. I similarly compared the proportion of single copy genes in each location and stage that were early and late losses (*POInT_RootLosses* versus *POInT_DrLosses*). For the anatomical tests, any gene expressed in the zygote was omitted from the analysis to avoid having the strong bias against ohnologs in this stage give rise to spurious associations.

Aanes et al., [73] have partitioned mRNAs from the early zebrafish embryo into three groups: genes expressed from inherited maternal transcripts, genes expressed from the embryo’s genome prior to the midblastula transition (pre-MTB) and genes expressed first in the zygotic stage (e.g., post-MTB). Using these gene lists, I compared the frequency of ohnologs and single-copy genes (*Dr_Ohno_all* vs *Dr_Sing_all* and *Dr_Ohno_POInT vs Dr_Sing_POInT*) in each, as well as the proportion of root losses and tip losses (*POInT_RootLosses* vs *POInT_DrLosses*) using a chi-square test in all cases (Table 1).

### GO analyses

I used the Gene List Analysis tool from the PANTHER classification system [version 13.1; 75] to find over or under-represented Gene Ontology (GO) terms associated with the surviving ohnologs (*Dr_Ohno_all* compared to *Dr_Sing_all* and *Dr_Ohno_POInT* to *Dr_Sing_POInT*) and the early versus late ohnolog losses (*POInT_RootLosses* compared to *POInT_DrLosses*). In each case, I asked whether there were any ontology terms that were significantly over or under-represented on the first list, using Fisher’s exact test with an FDR multiple test correction [75, 134]. Lists of all significantly enriched terms for all comparisons are given as S3 Table.

### Gene essentiality and the TGD

From ZFIN [72], I extracted all genes with known phenotypes, as well as the subset of those genes with phenotypes described as “lethal,” “dead” or “inviable:” hereafter I refer to this second set as the “essential genes.” I compared the proportion of phenotyped ohnologs in the essential list to the same proportion among the single copy genes. For comparative purposes, I obtained a list of essential mouse genes from the International Mouse Phenotyping Consortium [82, 83]. Using our orthology inference pipeline ORIS (ORthology Inference using Synteny), I inferred the gar orthologs of these mouse genes [135, 136], retrieving 10,644 gar genes with a mouse ortholog. For each gar gene with phenotype data in a mouse ortholog, we compared the proportion of genes with a surviving ohnolog in zebrafish that were essential when knocked out in mouse to the proportion of genes without a surviving zebrafish ohnolog pair that were essential in mouse (Table 2; other phenotype classes such as “subviable” were excluded).

### The TGD and the zebrafish metabolic network

I extracted an enzyme-centered metabolic network from the reconstruction of zebrafish metabolism published by Bekaert [84]. In this network nodes are biochemical reactions and edges connect pairs of nodes with a common metabolite. The 13 currency metabolites given by Bekaert [84] were excluded from the edge computation. Each reaction was linked to one or more Ensembl gene identifiers corresponding to genes encoding enzymes catalyzing that reaction.

To test for differences in network position between the products of ohnologs and single-copy genes, I compared the two groups for three statistics (see Results), using randomization to assess the statistical significance of any differences. To maintain the structure introduced by the WGD, all ohnolog pairs were reduced to a single entity, which was then assigned to all nodes that products of either of the two ohnologs appeared in. These merged ohnolog products were then randomized along with the products of the single copy genes, and the differences in the three statistics for each randomized network recomputed. If less than 5% of the randomized networks had a difference as large as that observed for the real data, I concluded that there was evidence for a difference between duplicated and unduplicated genes.

## Supporting information

S1 Dataset For each genome with the TGD, I show the synteny relationships seen in the estimated optimal ancestral order (tab-delimited text). In these files the symbol “<->” between a pair of genes indicates those genes are in synteny with each other, while “|” and “X” characters denote synteny breaks.
(PDF)

## Abbreviations

WGD: whole-genome duplication
TGD: teleost genome duplication
DCS: double conserved synteny
POInT: Polyploid Orthology Inference Tool
