# Supplementary material for: The lasting after-effects of an ancient polyploidy on the genomes of teleosts
Source: PLoS One. 2020 Apr 16;15(4):e0231356. doi: 10.1371/journal.pone.0231356 (PMC7161988; doi:10.1371/journal.pone.0231356)
Supplement: S1 Dataset — In these files the symbol “<->” between a pair of genes indicates those genes are in synteny with each other, while “|” and “X” characters denote synteny breaks. (PDF) [file pone.0231356.s001.pdf]

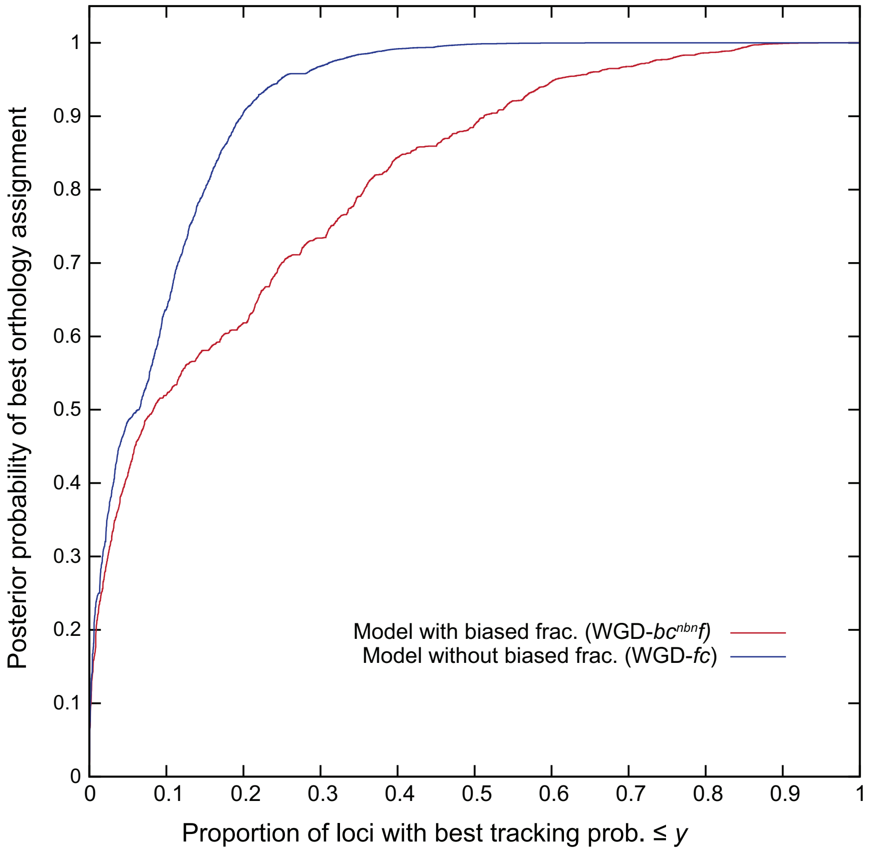

Supplemental Figure 1: Estimated confidence of POInT's orthology and subgenome assignments for models with and without biased fractionation (WGD-$bc^{nbn}f$ and WGD-*fc*, respectively: note that the WGD-*fc* model is simply the WGD-*bfc* model of Figure 2 with ε_1=ε_2=1.0). For each ancestral locus, I identified the orthology assignment with highest posterior probability, and sorted this probability across all pillars. Hence, the *x*-axis gives the proportion of pillars with posterior probabilities of their best orthology assignments ≤*y*. For the WGD-*fc* model, the posterior probabilities are multiplied by 2 to account for the subgenome degeneracy of the model. The higher posterior probabilities of the WGD-*fc* model can be understood as cases where the loss patterns clearly mark orthologous regions, but where the signal of bias in fractionation is weaker and hence consistent assignment to the more or less fractionated subgenome is less certain.

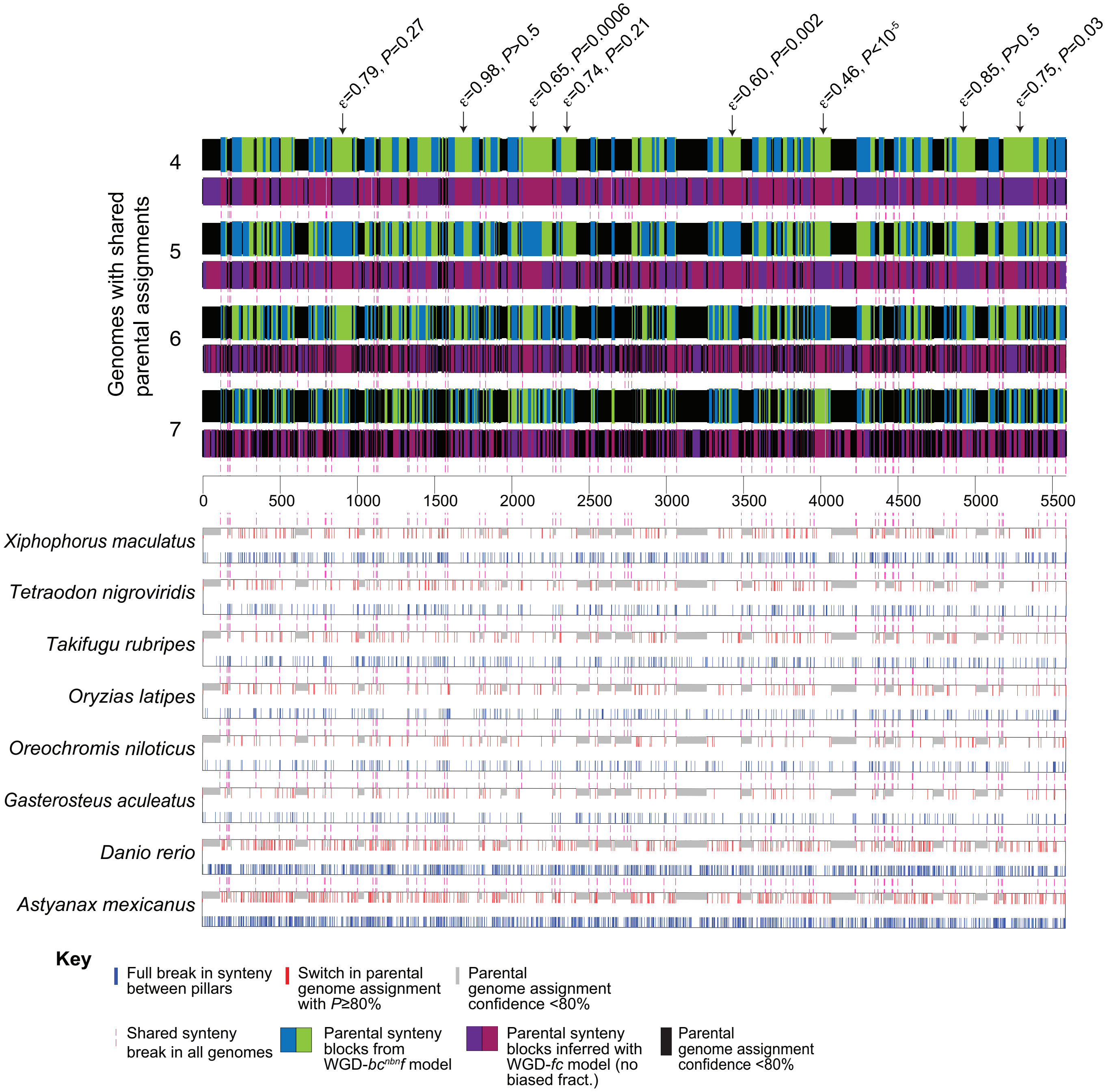

Supplemental Figure 2: Inferred subgenome assignments and synteny breaks across eight genomes. In the lower eight panels, I illustrate how often POInT's assignment of parental subgenome inferences change between successive pillars in each extant genome under the WGD-$bc^{nbn}f$ model. A red tick at position *i* corresponds where the assigned parents-of-origin changed relative to *i-1* with probability of ≥80%. Gray ticks correspond to positions immediately after a red tick where the confidence in the parental assignments is less than 80%. The blue ticks in the lower half of each block indicate positions where there is a double synteny break after position *i-1*. Locations where all 8 genomes have such double synteny breaks are shown as pink dotted lines. In the upper four pairs of panels are estimates of shared parental blocks across genomes for two different models. The colored block correspond to cases (from bottom to top) where at 7,6,5 or 4 genomes agree with confidence ≥80%. The blue/green blocks are computed from the WGD-$bc^{nbn}f$ model and indicate confidence not only in orthology across the genomes but also confidence in assigning groups of genes to the more or less fractionated subgenome. Red/purple

blocks are computed with the WGD-*fc* model. In this case, only orthology is considered, as the two sugenomes are degenerate. Narrower black regions have no position-to-position agreement in subgenome assignments. Note that most of the observed uncertainty corresponds to the more or less fractionated subgenome assignment (blue/green), while orthology assignments are generally of high confidence. Using the WGD-$bc^{nbn}f$ model, I also extracted the 8 blocks shared with >80% confidence across 4 genomes and with >100 pillars included and fit the WGD-*f* and WGD-*bf* to them. Above each, I report the estimated value of ε and the significance of the test of model WGD-*bf* offering a better fit than WGD-*f* (likelihood ratio test with 1 degree of freedom).

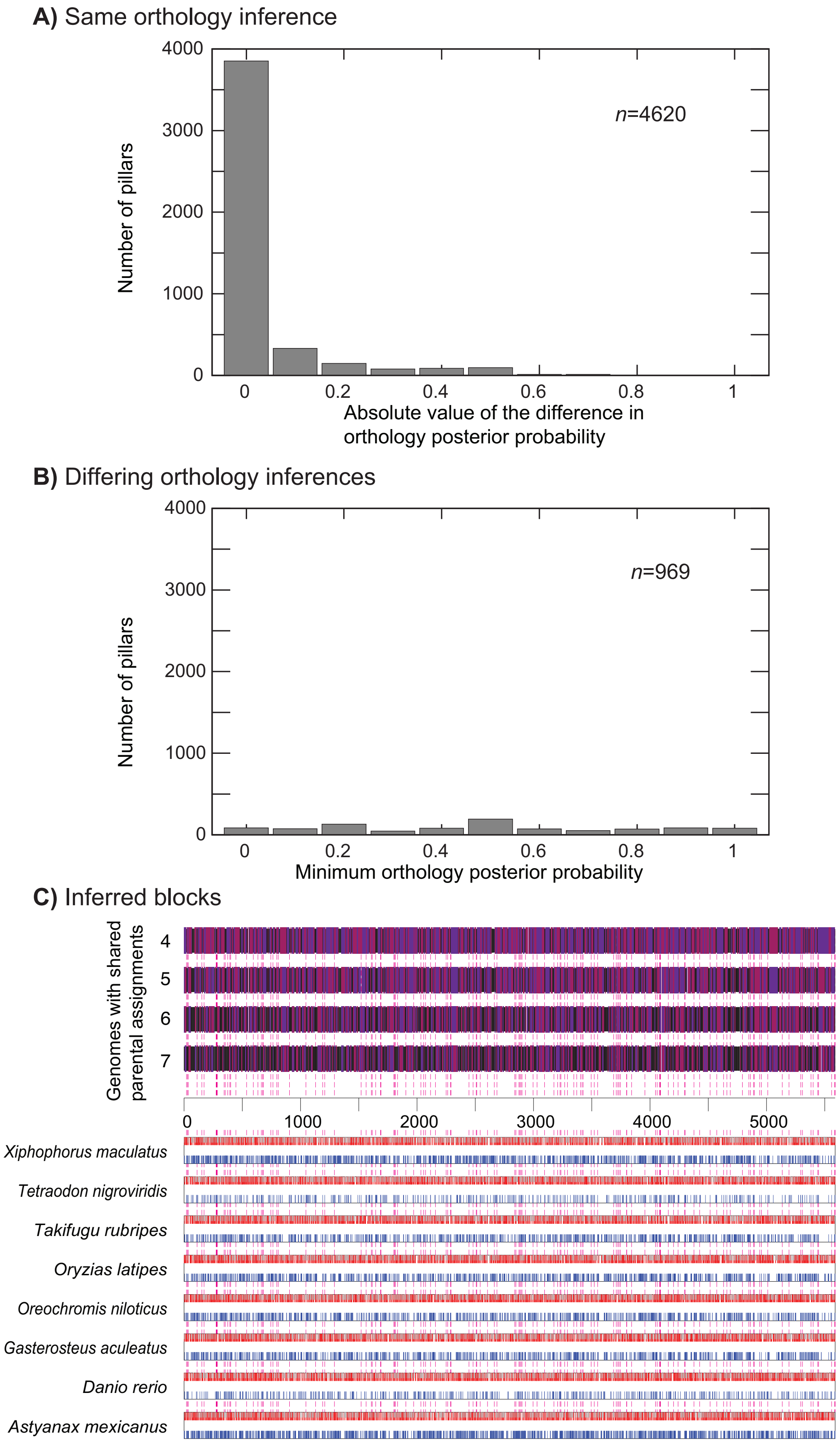

Supplemental Figure 3: The use of different inferred ancestral orders does not greatly alter POInT's orthology inferences. We compared the inferred orthology relationships from the order giving the maximum likelihood (Supplemental Table 2) to an order that minimized the sum of the number of breaks in the genomes of *D. rerio* and *T. nigroviridis* (Order *DR_TN*; see *Methods*). **A)** Distribution of the absolute value of the difference in probability of the best inferred orthology relationships for each pillar between the two pillar orders (*x*-axis) for all cases where the best orthology inference is the same for the two orders under the WGD-*f* model (*n*=4620). **B)** Distribution of the minimum of probability of the best inferred orthology relationships for each pillar between the two pillar orders (*x*-axis) for all cases where the best orthology inference in **not** the same for the two orders under the WGD-*f* model (*n*=969). **C)** Pattern of inferred blocks and synteny breaks for the pillars in the order *DR_TN* (see Supplemental Figure 2).

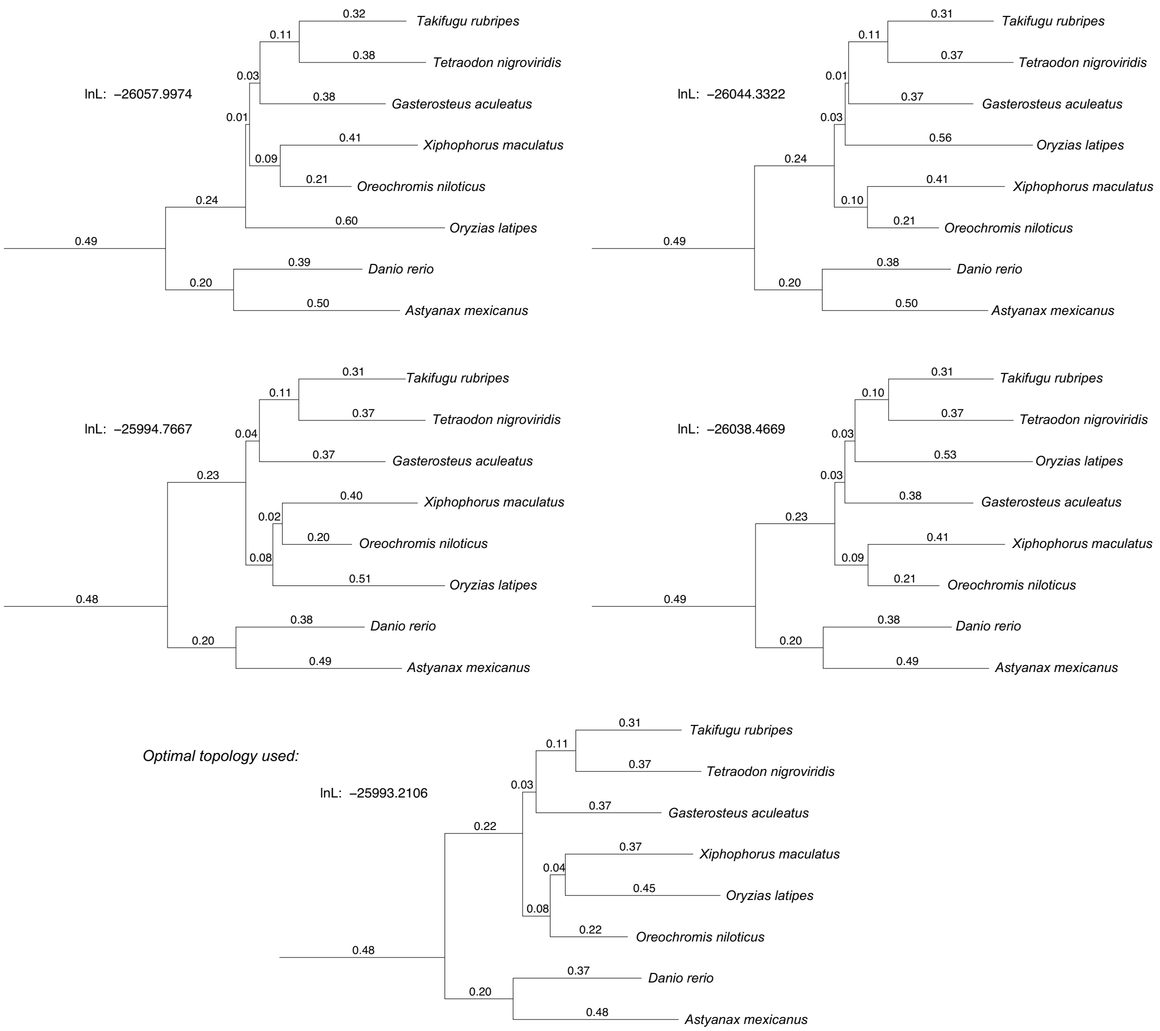

Supplemental Figure 4: Alternative phylogenetic topologies and the TGD. I tested 4 other topologies in addition to that of Near et al., (Near et al. 2012) using the optimal DCS block order and the WGD-$bc^{nbn}f$ model in POInT: shown are the induced branch lengths for these topologies as well as the corresponding model parameter estimates. For reference, the topology at the bottom is that of Near et al., used for all other analyses.

Supplemental Table 1: Effects of the estimated ancestral pillar order on parameter estimates from POInT.

Optimization Method[a]	Breaks[b]	lnL[c]	γ[d]	ε[d]	δ[d]	θ[d]
	14342	-26240.049	0.075	0.676	0.342	0.0089
	14693	-26170.596	0.074	0.669	0.339	0.0085
	14787	-26188.226	0.075	0.684	0.343	0.0084
	14965[e]	*-26080.462*	*0.074*	*0.657*	*0.341*	*0.0079*
Global_Break_Opt	15148	-26178.494	0.076	0.683	0.352	0.0076
	15966	-26272.204	0.076	0.728	0.382	0.0076
	16323	-26272.011	0.075	0.673	0.361	0.0073
	16766	-26323.416	0.074	0.680	0.379	0.0074
	17523	-26265.175	0.076	0.723	0.407	0.0066
	21656	-27092.183	0.071	0.634	0.416	0.0052
	13852	-26226.784	0.077	0.701	0.370	0.0097
	13898	-26203.132	0.078	0.711	0.370	0.0087
	13937	-26103.825	0.077	0.685	0.356	0.0084
	13974	-26169.419	0.077	0.704	0.361	0.0087
Greedy_Opt	14086	-26136.803	0.079	0.751	0.383	0.008
	14370[f]	***-25993.112***	***0.077***	***0.701***	***0.371***	***0.0074***
	14698	-26114.818	0.076	0.681	0.370	0.0084
	14847	-26147.026	0.076	0.695	0.377	0.0086
	15105	-26207.243	0.073	0.634	0.354	0.0079
	15253	-26175.667	0.075	0.680	0.369	0.008
	13658	-26362.214	0.074	0.61	0.326	0.01
	13695	-26290.942	0.076	0.662	0.343	0.0091
	13772	-26314.732	0.078	0.687	0.357	0.0091
	13897	-26324.514	0.079	0.669	0.345	0.0083
Naïve_Opt	13912	-26323.322	0.081	0.722	0.365	0.0084
	13945	-26238.085	0.080	0.699	0.358	0.0081
	13978	-26257.832	0.077	0.661	0.344	0.0088
	14002	-26279.572	0.079	0.708	0.363	0.0086
	14010[g]	*-26161.701*	*0.078*	*0.674*	*0.351*	*0.0084*
	14122	-26336.625	0.076	0.639	0.333	0.0088
D. rerio/T. nigroviridis Opt	18511	-27339.695	0.076	0.575	0.382	0.0070

a: Four methods for estimating the ancestral pillar order at the TGD were used: See *Methods* for details.

b: Number of synteny breaks in the estimated order (see *Methods*).

c: ln-likelihood of the pillar data under the assumed order and the WGD-$bc^{nbn}f$ model (Figure 2).

d: Parameter estimates for the WGD-$bc^{nbn}f$ model: γ=fixation rate, ε=strength of biased fractionation, δ=rate of duplicates entering the converging state, θ=estimated pillar-to-pillar probability of a change in orthology assignment. See Figure 2.

e: Order with the largest lnL, Global_Break_Opt

f: Order with the **overall** largest lnL across all three optimization methods.

g: Order with the largest lnL, Naïve_Opt

Supplemental Table 2: Inference of Double-Conserved Synteny (DCS) blocks from eight teleost genomes by reference to the spotted gar genome.

Species	Total genes in dataset[a]	% of genes with 1:1/1:2 homology to gar[b]	% genes in synteny blocks[c]	% genes in synteny blocks w/ >5 genes[d]	# of genes not in synteny blocks with ambig. homology[e]
Astyanax mexicanus	6707	71.4	94.0	80.2	116
Danio rerio	6849	69.3	95.3	85.1	97
Gasterosteus aculeatus	6799	69.6	99.2	98.0	22
Oreochromis niloticus	6936	69.6	99.4	97.8	13
Oryzias latipes	6601	69.9	99.3	97.3	12
Takifugu rubripes	6725	70.4	99.0	96.6	34
Tetraodon nigroviridis	6660	70.0	98.7	96.4	41
Xiphophorus maculatus	6685	70.1	98.0	93.4	41

a: Number of genes from the genome placed into the shared DCS blocks used for POInT analyses (see *Methods*).

b: Percent of cases in the genome in question where genes in the final dataset from that genome match one and only one gene in the gar genome and the gar gene in question matches at most two genes in that genome.

c: Percentage of genes from the genome in question where that gene is in a synteny block with at least one other gene using the optimal ancestral order from Supplemental Table 2.

d: Percentage of genes from the genome in question where that gene is in a synteny block with at least four other genes (blocks of size 5) using the optimal ancestral order from Supplemental Table 2.

e: Number of genes in the dataset analyzed which are not in synteny blocks in the optimal order that also have some ambiguity in their homology relationships: either a teleost gene with multiple gar homologs or where the gar gene in question has >2 homologs in this genome.

Supplemental Table 3: Differentially abundant GO terms for Ohnologs and single-copy genes from the TGD

Comparison[a]	GO Hierarchy	GO Term	# in Ohnolog set[b]	Ohnolog enrichment[c]	P-value[d]
Dr_Ohno_all/ Dr_Sing_all[e]	Molecular Function	channel regulator activity	4	9.21	1.32E-04
		carbohydrate phosphatase activity	2	7.89	2.85E-02
		phosphatase activity	115	1.67	3.98E-03
		cyclic nucleotide-gated ion channel activity	2	7.89	2.79E-02
		ligand-gated ion channel activity	78	2.8	3.95E-09
		ion channel activity	186	2.43	1.26E-14
		transmembrane transporter activity	468	1.82	7.59E-15
		transporter activity	555	1.73	1.33E-14
		voltage-gated calcium channel activity	8	7.56	1.49E-06
		cation channel activity	58	2.86	2.94E-07
		cation transmembrane transporter activity	139	1.65	1.96E-03
		voltage-gated ion channel activity	51	2.48	1.27E-04
		glutamate receptor activity	12	7.45	3.70E-09
		receptor activity	610	1.32	4.79E-04
		phosphatase inhibitor activity	4	5.26	2.19E-02
		antigen binding	13	4.05	7.02E-04
		binding	2653	1.18	1.14E-06
		gap junction channel activity	14	3.19	9.12E-03
		voltage-gated potassium channel activity	37	3.06	1.26E-05
		kinase inhibitor activity	14	2.63	4.10E-02
		kinase activity	347	1.42	8.31E-04
		lipid transporter activity	25	2.53	9.15E-03
		ATPase activity, coupled to transmembrane movement of substances	38	2.35	2.67E-03
		actin binding	72	2.34	2.06E-05
		cytoskeletal protein binding	194	1.56	1.21E-03
		protein binding	1496	1.3	1.62E-08
		calmodulin binding	51	2.32	4.23E-04
		adenylate cyclase activity	66	1.99	2.12E-03
		sequence-specific DNA binding RNA polymerase II transcription factor activity	137	1.81	1.36E-04
		sequence-specific DNA binding transcription factor activity	410	1.35	2.41E-03
		calcium ion binding	117	1.62	9.13E-03
		signal transducer activity	524	1.54	3.14E-08
		GTPase activity	220	1.41	1.33E-02
		protein kinase activity	227	1.38	2.09E-02
		receptor binding	422	1.27	1.94E-02
		Unclassified	5234	0.89	2.77E-08
		oxidoreductase activity	345	0.71	1.07E-02
		RNA binding	232	0.5	7.27E-05
		cysteine-type peptidase activity	71	0.48	4.94E-02
		isomerase activity	66	0.36	9.09E-03
		nuclease activity	100	0.29	9.51E-05
		helicase activity	48	0.27	9.10E-03
		single-stranded DNA binding	31	0.25	4.35E-02
		structural constituent of ribosome	89	0.18	3.35E-06
		nucleotidyltransferase activity	47	0.06	6.82E-05
		RNA methyltransferase activity	25	< 0.01	2.74E-03
		methyltransferase activity	72	0.22	1.68E-04
		hydro-lyase activity	28	< 0.01	1.20E-03
		aminoacyl-tRNA ligase activity	32	< 0.01	5.47E-04
		ligase activity	148	0.57	1.10E-02
		peroxidase activity	16	< 0.01	3.59E-02
		damaged DNA binding	21	< 0.01	1.02E-02
Dr_Ohno_POInT/ Dr_Sing_POInT[f]	Molecular Function	channel regulator activity	10	8.35	1.40E-02
		glutamate receptor activity	31	7.39	2.10E-06
		voltage-gated calcium channel activity	15	6.26	4.85E-03
		cation channel activity	49	3.27	1.98E-05
		ion channel activity	116	2.52	2.03E-08
		transmembrane transporter activity	215	1.75	2.50E-07
		transporter activity	244	1.7	1.87E-07
		cation transmembrane transporter activity	59	1.64	3.52E-02
		voltage-gated ion channel activity	27	2.25	3.10E-02

		translation elongation factor activity	11	4.59	3.03E-02
		RNA binding	21	0.41	1.43E-03
		binding	739	1.15	7.64E-03
		translation regulator activity	25	2.78	1.33E-02
		ATPase activity, coupled to transmembrane movement of substances	24	3.08	8.81E-03
		ligand-gated ion channel activity	63	2.84	7.31E-06
		adenylate cyclase activity	39	2.03	1.74E-02
		actin binding	38	1.98	2.35E-02
		protein binding	479	1.27	3.80E-04
		sequence-specific DNA binding RNA polymerase II transcription factor activity	59	1.76	1.78E-02
		sequence-specific DNA binding transcription factor activity	133	1.41	1.97E-02
		signal transducer activity	157	1.61	2.88E-04
		kinase activity	146	1.46	7.74E-03
		Unclassified	993	0.88	1.62E-04
		oxidoreductase activity	55	0.65	2.91E-02
		isomerase activity	4	0.23	1.38E-02
		structural constituent of ribosome	4	0.16	3.45E-04
		nuclease activity	2	0.1	6.77E-04
		RNA methyltransferase activity	0	< 0.01	1.93E-02
		methyltransferase activity	2	0.1	2.15E-04
		hydro-lyase activity	0	< 0.01	1.50E-02
		aminoacyl-tRNA ligase activity	0	< 0.01	1.87E-02
		peroxidase activity	0	< 0.01	2.79E-02
		antioxidant activity	0	< 0.01	6.03E-03
		single-stranded DNA binding	0	< 0.01	1.43E-02
		nucleotidyltransferase activity	0	< 0.01	1.40E-03
		DNA helicase activity	0	< 0.01	1.80E-02
		helicase activity	2	0.16	2.27E-02
POInT_RootLosses /POInT_DrLosses[g]	Molecular Function	No Significantly differentially abundant terms			
POInT_AllOhnologs/POInT_AllSingle.[h]	Molecular Function	hydrolase activity, acting on acid anhydrides, in phosphorus-containing anhydrides	23	9.82	4.44E-02
		hydrolase activity, acting on acid anhydrides	23	9.82	3.70E-02
		Unclassified	75	113.02	1.31E-03
Dr_Ohno_all/ Dr_Sing_all[e]	Biological Process	gluconeogenesis	11	4.82	6.36E-03
		primary metabolic process	933	0.89	2.01E-03
		asymmetric protein localization	8	4.21	3.43E-02
		localization	500	1.13	4.48E-02
		blood circulation	16	4.21	2.62E-03
		system process	353	2	5.00E-21
		single-multicellular organism process	549	1.93	6.42E-31
		multicellular organismal process	550	1.93	6.30E-31
		complement activation	20	4.05	5.98E-04
		response to stimulus	699	1.32	2.42E-09
		muscle organ development	20	4.05	5.82E-04
		system development	163	2.06	1.27E-10
		developmental process	483	1.48	1.63E-11
		B cell mediated immunity	20	4.05	5.67E-04
		muscle contraction	79	3.71	8.17E-13
		cell recognition	24	3.51	4.85E-04
		cellular process	2002	1.14	4.72E-10
		defense response to bacterium	20	3.29	2.61E-03
		spermatogenesis	17	3.19	7.33E-03
		neuron-neuron synaptic transmission	54	2.9	8.22E-07
		synaptic transmission	180	1.93	5.20E-10
		cell-cell signaling	240	1.93	3.51E-13
		cell communication	840	1.47	6.24E-20
		response to biotic stimulus	23	2.88	2.70E-03
		synaptic vesicle exocytosis	31	2.4	3.13E-03
		neurotransmitter secretion	45	1.97	4.05E-03
		neurological system process	289	1.84	4.15E-14
		embryo development	50	2.16	5.88E-04
		behavior	27	2.15	1.35E-02
		nervous system development	130	2.12	3.71E-09
		negative regulation of apoptotic process	41	2	4.94E-03
		regulation of biological process	715	1.42	2.34E-14
		biological regulation	877	1.42	5.40E-18
		phagocytosis	48	1.97	2.83E-03

		endocytosis	107	1.62	6.53E-04
		protein phosphorylation	38	1.92	1.11E-02
		cyclic nucleotide metabolic process	50	1.85	4.53E-03
		nucleobase-containing compound metabolic process	498	0.82	2.59E-04
		G-protein coupled receptor signaling pathway	116	1.84	5.77E-06
		cell surface receptor signaling pathway	335	1.51	1.33E-08
		signal transduction	714	1.45	7.14E-16
		MAPK cascade	126	1.81	3.46E-06
		intracellular signal transduction	393	1.58	4.71E-12
		cellular component morphogenesis	157	1.78	3.48E-07
		anatomical structure morphogenesis	53	1.62	2.46E-02
		cellular component organization	445	1.14	4.60E-02
		cytokinesis	50	1.73	1.33E-02
		cell differentiation	185	1.7	2.48E-07
		mesoderm development	105	1.68	2.94E-04
		sensory perception	61	1.57	2.18E-02
		regulation of phosphate metabolic process	173	1.52	9.47E-05
		phosphate-containing compound metabolic process	462	1.27	6.57E-05
		regulation of catalytic activity	126	1.52	1.32E-03
		regulation of molecular function	152	1.53	3.01E-04
		response to endogenous stimulus	89	1.47	1.49E-02
		locomotion	94	1.43	2.05E-02
		anion transport	81	1.41	4.51E-02
		ion transport	128	1.43	5.61E-03
		homeostatic process	101	1.41	2.25E-02
		cytoskeleton organization	109	1.36	3.10E-02
		Unclassified	1403	0.83	3.01E-13
		response to stress	83	0.66	2.63E-03
		protein targeting	22	0.57	4.64E-02
		DNA replication	14	0.47	2.35E-02
		DNA metabolic process	26	0.34	2.77E-08
		purine nucleobase metabolic process	6	0.37	4.53E-02
		RNA catabolic process	5	0.35	4.67E-02
		RNA metabolic process	230	0.69	6.20E-07
		cell-matrix adhesion	6	0.3	1.10E-02
		regulation of sequence-specific DNA binding transcription factor activity	2	0.19	3.55E-02
		DNA repair	5	0.12	7.42E-09
		rRNA metabolic process	3	0.11	1.58E-06
		tRNA metabolic process	3	0.1	2.99E-07
		tRNA aminoacylation for protein translation	1	0.08	2.88E-03
		translation	11	0.22	6.60E-08
		cytokine production	0	< 0.01	4.66E-02
		mRNA polyadenylation	0	< 0.01	4.68E-02
		mRNA 3'-end processing	0	< 0.01	8.68E-03
		mRNA processing	33	0.6	2.34E-02
		pteridine-containing compound metabolic process	0	< 0.01	4.62E-02
		nitrogen compound metabolic process	482	0.84	2.35E-03
		mitochondrial translation	0	< 0.01	3.12E-02
		mitochondrion organization	8	0.34	7.45E-03
		fatty acid beta-oxidation	0	< 0.01	4.56E-02
Dr_Ohno_POInT/Dr_Sing_POInT	Biological Process	blood circulation	10	5.56	2.82E-02
		system process	254	2.27	7.43E-17
		single-multicellular organism process	383	2.15	5.67E-24
		multicellular organismal process	384	2.16	7.49E-24
		muscle contraction	58	5.1	4.18E-10
		synaptic vesicle exocytosis	23	3.84	1.44E-03
		neurotransmitter secretion	35	2.54	3.16E-03
		neurological system process	214	2.13	1.62E-12
		neuron-neuron synaptic transmission	47	3.41	6.88E-06
		synaptic transmission	130	2.09	1.98E-07
		cell-cell signaling	161	1.93	1.33E-07
		cell communication	564	1.55	1.34E-14
		cellular process	1323	1.16	1.51E-07
		sensory perception	54	2.65	5.74E-05
		nervous system development	98	2.27	1.21E-06
		system development	119	2.31	3.87E-08
		developmental process	329	1.46	1.71E-06

		G-protein coupled receptor signaling pathway	65	2.21	1.81E-04
		cell surface receptor signaling pathway	218	1.63	1.81E-06
		signal transduction	490	1.58	2.35E-13
		cellular calcium ion homeostasis	33	2.12	2.02E-02
		homeostatic process	68	1.77	5.85E-03
		biological regulation	531	1.45	1.73E-10
		cyclic nucleotide metabolic process	39	1.97	2.10E-02
		nucleobase-containing compound metabolic process	338	0.85	3.53E-02
		primary metabolic process	620	0.88	1.58E-02
		transmembrane receptor protein tyrosine kinase signaling pathway	40	1.91	2.46E-02
		cellular component morphogenesis	104	1.89	7.12E-05
		cell differentiation	132	1.88	5.44E-06
		locomotion	66	1.87	3.09E-03
		MAPK cascade	95	1.76	7.73E-04
		intracellular signal transduction	281	1.67	6.47E-09
		regulation of catalytic activity	85	1.73	2.19E-03
		regulation of molecular function	105	1.79	2.53E-04
		mesoderm development	70	1.65	1.75E-02
		endocytosis	66	1.62	2.32E-02
		cellular component movement	89	1.62	7.40E-03
		regulation of phosphate metabolic process	123	1.58	1.63E-03
		phosphate-containing compound metabolic process	335	1.29	1.50E-03
		regulation of biological process	422	1.45	6.26E-08
		regulation of nucleobase-containing compound metabolic process	94	1.47	2.64E-02
		Unclassified	761	0.84	7.28E-06
		response to stress	38	0.52	1.66E-03
		response to stimulus	411	1.38	2.66E-06
		mRNA processing	17	0.46	1.62E-02
		RNA metabolic process	146	0.66	5.56E-05
		DNA replication	7	0.34	2.50E-02
		DNA metabolic process	15	0.29	7.57E-06
		DNA repair	5	0.2	6.51E-04
		cell-matrix adhesion	3	0.18	6.40E-03
		RNA catabolic process	2	0.16	1.89E-02
		rRNA metabolic process	2	0.1	2.12E-04
		tRNA aminoacylation for protein translation	0	< 0.01	1.53E-02
		translation	8	0.22	1.36E-05
		protein metabolic process	194	0.78	1.29E-02
		tRNA metabolic process	0	< 0.01	3.36E-05
POInT_RootLosses /POInT_DrLosses[g]	Biological Process	synaptic transmission	26	0.27	3.74E-02
		cell-cell signaling	33	0.28	2.03E-02
POInT_AllOhnologs/POInT_AllSingle.[h]	Biological Process	positive regulation of neuron projection development	4	0.08	1.14E-02
		regulation of cell differentiation	6	0.83	2.99E-02
		regulation of developmental process	6	0.91	3.35E-02
		cellular process	121	85.2	1.43E-03
		multicellular organismal process	26	12.47	3.33E-02
		positive regulation of neuron differentiation	6	0.08	4.48E-04
		positive regulation of neurogenesis	6	0.17	1.12E-03
		positive regulation of cell development	6	0.25	1.88E-03
		positive regulation of cell differentiation	6	0.33	3.15E-03
		protein kinase B signaling	4	0.08	1.09E-02
		intracellular signal transduction	22	9.91	3.22E-02
		cell communication	22	6.85	1.46E-03
		synaptic vesicle endocytosis	4	0.08	9.93E-03
		establishment of localization	37	16.92	2.82E-03
		localization	48	27.24	1.57E-02
		endocytosis	14	2.06	6.17E-04
		vesicle-mediated transport	22	8.59	1.17E-02
		transport	37	16.92	2.60E-03
		synaptic vesicle cycle	5	0.08	1.87E-03
		insulin receptor signaling pathway	3	0.08	4.59E-02
		cellular response to insulin stimulus	5	0.25	8.88E-03
		cellular response to peptide hormone stimulus	5	0.41	2.09E-02
		cellular response to peptide	5	0.41	2.15E-02
		cellular response to endogenous stimulus	7	1.32	3.37E-02
		response to endogenous stimulus	7	1.32	3.44E-02

		glycogen metabolic process	4	0.17	2.39E-02
		cellular glucan metabolic process	4	0.17	2.22E-02
		carbohydrate metabolic process	12	3.38	2.09E-02
		cellular polysaccharide metabolic process	4	0.25	3.37E-02
		generation of precursor metabolites and energy	4	0.25	3.29E-02
		gluconeogenesis	4	0.17	2.30E-02
		glucose metabolic process	4	0.25	3.21E-02
		carbohydrate biosynthetic process	4	0.08	1.04E-02
		organic substance biosynthetic process	16	5.45	1.95E-02
		phagocytosis	4	0.17	2.15E-02
		ATP biosynthetic process	4	0.25	3.45E-02
		purine ribonucleoside triphosphate biosynthetic process	4	0.25	3.14E-02
		phosphorus metabolic process	8	1.73	3.35E-02
		purine nucleoside triphosphate biosynthetic process	4	0.25	3.53E-02
		pyruvate metabolic process	4	0.33	4.83E-02
		membrane invagination	10	0.91	6.82E-04
		membrane organization	17	3.63	4.33E-04
		vesicle budding from membrane	10	0.91	4.54E-04
		cytokinesis	6	0.99	4.44E-02
		vesicle fusion to plasma membrane	8	1.57	2.51E-02
		membrane fusion	8	1.9	4.68E-02
		plasma membrane fusion	8	1.65	3.12E-02
		chemical synaptic transmission	15	3.88	3.95E-03
		anterograde trans-synaptic signaling	15	3.88	3.29E-03
		trans-synaptic signaling	15	3.88	3.70E-03
		synaptic signaling	15	3.88	3.48E-03
		cell-cell signaling	22	6.6	1.01E-03
		intracellular protein transport	23	9.91	2.13E-02
		organic substance transport	8	1.49	2.06E-02
		Unclassified	76	103.36	2.45E-02
Dr_Ohno_all/ Dr_Sing_all[e]	Cellular Compartment	plastid	4	10.52	4.55E-02
		organelle	687	0.8	1.44E-07
		basal part of cell	5	6.58	4.30E-02
		cell part	1106	0.89	2.59E-04
		postsynaptic membrane	24	4.51	2.12E-05
		membrane	694	1.42	9.64E-14
		synapse	63	2.55	1.42E-06
		axon	32	3.66	1.05E-05
		neuron projection	122	1.92	5.43E-07
		cell projection	147	1.59	5.02E-05
		immunoglobulin complex	20	3.51	7.23E-04
		macromolecular complex	406	0.88	4.42E-02
		presynaptic membrane	12	2.63	4.28E-02
		actin cytoskeleton	96	2.53	2.40E-09
		cytoskeleton	160	1.34	7.54E-03
		dendrite	53	2.02	4.93E-04
		plasma membrane	583	1.71	1.41E-23
		integral to membrane	342	1.46	1.20E-07
		extracellular space	128	1.42	4.43E-03
		extracellular region	166	1.25	4.27E-02
		nucleoplasm	55	0.7	3.56E-02
		nucleus	288	0.7	1.21E-07
		cytosol	81	0.64	5.10E-04
		cytoplasm	653	0.89	7.75E-03
		intracellular	1013	0.86	4.74E-06
		mitochondrion	52	0.57	5.38E-04
		chromosome	16	0.41	1.11E-03
		ribosome	8	0.21	2.79E-06
		ribonucleoprotein complex	26	0.26	5.49E-14
		nuclear envelope	4	0.21	1.06E-03
		nucleolus	3	0.1	2.39E-07
Dr_Ohno_POInT/ Dr_Sing_POInT[f]	Cellular Compartment	postsynaptic membrane	23	4.8	2.68E-04
		membrane	448	1.41	1.89E-07
		synapse	55	2.96	9.61E-06
		presynaptic membrane	10	4.17	3.65E-02
		axon	22	4.08	1.06E-03
		neuron projection	78	2.1	1.08E-04
		cell projection	91	1.65	2.59E-03

		cell part	745	0.91	2.68E-02
		dendrite	40	2.9	2.34E-04
		actin cytoskeleton	57	2.44	1.22E-04
		organelle	468	0.82	3.44E-04
		plasma membrane	368	1.68	5.47E-12
		integral to membrane	217	1.5	8.00E-05
		intracellular	690	0.88	3.23E-03
		nucleoplasm	41	0.63	3.76E-02
		nucleus	202	0.74	7.21E-04
		mitochondrial inner membrane	6	0.33	2.96E-02
		mitochondrion	42	0.64	3.80E-02
		ribosome	6	0.23	7.64E-04
		ribonucleoprotein complex	16	0.23	1.39E-09
		macromolecular complex	260	0.81	9.44E-03
		nucleolus	2	0.08	1.13E-05
POInT_RootLosses /POInT_DrLosses[g]	Cellular Compartment	neuron projection	14	0.21	4.59E-02
POInT_ AllOhnologs/POInT_AllSingle.[h]	Cellular Compartment	axon	8	0.25	5.53E-05
		neuron projection	10	1.9	8.52E-03
		cell part	112	78.92	4.01E-03
		cell	112	79.01	3.04E-03
		neuron part	17	3.72	3.77E-04
		synaptic vesicle	5	0.25	7.63E-03
		presynapse	7	0.91	1.20E-02
		intracellular	93	67.2	3.35E-02

a: Ohnolog and single-copy gene sets used for comparisons (See *Methods*).

b: Number of genes in the ohnolog set with the specified Go term (see **e** and **f** for totals).

c: Enrichment of the specified term in the ohnolog set relative to the single-copy set (values less than 1.0 represent GO terms seen less frequently among the surviving ohnolog pairs).

d: FDR-corrected *P*-value for the test of the hypothesis of equal proportions of genes in the ohnolog and single-copy gene sets annotated with the term in question.

e: See *Methods* for set definitions: *Dr_Ohno_all* includes 3994 genes and *Dr_Sing_all* contains 11,097 genes.

f: See *Methods* for set definitions: *Dr_Ohno_POInT* includes 2520 genes and *Dr_Sing_POInT* contains 4329 genes.

g: See *Methods* for set definitions: *POInT_RootLosses* includes 1880 genes and *POInT_DrLosses* contains 245 genes.

h: Compares the zebrafish annotation of ohnologs present in all eight genomes (n=132 pairs) with zebrafish annotation of genes present only in single copy in all genomes ($n=3323$, *Methods*).

Supplemental Table 4: Developmental time points and anatomical regions with more or fewer ohnologs present than expected.

Anatomical Region	Dr_Ohno_ POInT: #Ohnologs/ Total (Exp.) [a]	FDR-corrected P-value	Dr_Ohno_All: #Ohnologs/ Total (Exp.) [a]	FDR-corrected P-value	POInT AllOhnologs/ Total (Exp.)[a,b]	FDR-corrected P-value
Blastula_EVL	10/24 (9.4)	1	14/49 (13.7)	1	4/16 (1.3)	0.75
Blastula_YSL	20/66 (26)	0.738	34/140 (39.1)	0.77	2/33 (2.7)	1
Blastula_margin	4/14 (5.5)	1	6/28 (7.8)	0.97	0/7 (0.6)	1
Blastula_proliferativeregion	2/4 (1.6)	1	4/16 (4.5)	1	0/2 (0.2)	1
Gastrula_adaxialcell	19/44 (17.3)	1	28/91 (25.4)	0.97	1/20 (1.7)	1
Gastrula_anterioraxialhypoblast	3/13 (5.1)	0.992	6/25 (7)	1	0/8 (0.7)	1
Gastrula_axis	11/32 (12.6)	1	18/61 (17)	1	0/16 (1.3)	1
Gastrula_centralnervoussystem	63/150 (59.1)	1	*93/251 (70.1)*	*0.02*	6/58 (4.8)	1
Gastrula_ectoderm	3/8 (3.1)	1	5/20 (5.6)	1	0/3 (0.2)	1
Gastrula_endoderm	3/10 (3.9)	1	7/26 (7.3)	1	0/5 (0.4)	1
Gastrula_forebrainneuralkeel	3/7 (2.8)	1	7/21 (5.9)	1	2/4 (0.3)	0.75
Gastrula_forerunnercellgroup	4/10 (3.9)	1	7/32 (8.9)	0.97	0/6 (0.5)	1
Gastrula_head	11/28 (11)	1	14/52 (14.5)	1	0/14 (1.2)	1
Gastrula_hindbrainneuralplate	6/16 (6.3)	1	11/33 (9.2)	0.97	1/8 (0.7)	1
Gastrula_integument	19/40 (15.7)	1	29/80 (22.4)	0.38	4/20 (1.7)	0.96
Gastrula_mesoderm	10/21 (8.3)	1	18/50 (14)	0.59	1/10 (0.8)	1
Gastrula_midbrainneuralkeel	4/14 (5.5)	1	4/22 (6.1)	0.85	2/7 (0.6)	1
Gastrula_neuralkeel	0/3 (1.2)	1	1/9 (2.5)	0.86	0/2 (0.2)	1
Gastrula_neuralplate	22/57 (22.4)	1	31/111 (31)	1	2/29 (2.4)	1
Gastrula_notochord	38/90 (35.4)	1	57/172 (48.1)	0.42	6/47 (3.9)	1
Gastrula_opticprimordium	6/12 (4.7)	1	9/28 (7.8)	1	0/5 (0.4)	1
Gastrula_oticplacode	16/44 (17.3)	1	28/108 (30.2)	1	2/27 (2.2)	1
Gastrula_paraxialmesoderm	6/12 (4.7)	1	10/23 (6.4)	0.42	1/5 (0.4)	1
Gastrula_periderm	16/40 (15.7)	1	26/93 (26)	1	5/26 (2.2)	0.82
Gastrula_polster	20/45 (17.7)	1	28/90 (25.1)	0.97	1/21 (1.7)	1
Gastrula_segmentalplate	8/27 (10.6)	1	17/62 (17.3)	1	1/15 (1.2)	1
Gastrula_tailbud	13/36 (14.2)	1	21/70 (19.6)	1	2/16 (1.3)	1
Gastrula_trunk	2/8 (3.1)	1	4/17 (4.7)	1	1/7 (0.6)	1
Gastrula_ventralmesoderm	18/46 (18.1)	1	32/83 (23.2)	0.15	0/22 (1.8)	1
Segmentation_Kupffersvesicle	2/11 (4.3)	0.850	8/44 (12.3)	0.51	0/8 (0.7)	1
Segmentation_alarplatemidbrainregion	10/41 (16.1)	0.608	15/82 (22.9)	0.24	0/24 (2)	1
Segmentation_artery	7/16 (6.3)	1	13/32 (8.9)	0.43	1/8 (0.7)	1
Segmentation_axialvasculature	9/13 (5.1)	0.548	13/29 (8.1)	0.24	1/3 (0.2)	1
Segmentation_ basalplatemidbrainregion	13/35 (13.8)	1	22/69 (19.3)	0.97	2/16 (1.3)	1
Segmentation_blood	8/24 (9.4)	1	13/42 (11.7)	1	1/15 (1.2)	1
Segmentation_brain	*106/209 (82.3)*	*0.029*	*147/405 (113.2)*	*0.003*	10/74 (6.2)	0.96
Segmentation_cardiovascularsystem	7/12 (4.7)	0.878	11/21 (5.9)	0.11	0/3 (0.2)	1
Segmentation_cerebellum	14/33 (13)	1	22/67 (18.7)	0.86	3/13 (1.1)	0.96
Segmentation_cranialganglion	36/74 (29.1)	0.698	*53/134 (37.4)*	*0.03*	5/24 (2)	0.75
Segmentation_diencephalon	*67/126 (49.6)*	*0.035*	*100/248 (69.3)*	*0.001*	6/44 (3.7)	1

Segmentation_dorsalaorta	5/9 (3.5)	1	8/18 (5)	0.50	0/2 (0.2)	1
Segmentation_epiphysis	*56/102 (40.2)*	*0.035*	*82/196 (54.8)*	*0.001*	8/39 (3.2)	0.75
Segmentation_fin	6/21 (8.3)	1	14/42 (11.7)	0.97	0/14 (1.2)	1
Segmentation_floorplate	8/21 (8.3)	1	14/57 (15.9)	1	2/14 (1.2)	1
Segmentation_forebrain	22/68 (26.8)	0.878	41/136 (38)	0.97	1/31 (2.6)	1
Segmentation_gut	19/66 (26)	0.673	38/150 (41.9)	0.97	3/37 (3.1)	1
Segmentation_hatchinggland	11/29 (11.4)	1	19/64 (17.9)	1	2/15 (1.2)	1
Segmentation_headmesenchyme	7/16 (6.3)	1	12/38 (10.6)	1	1/9 (0.7)	1
Segmentation_heartrudiment	10/24 (9.4)	1	17/45 (12.6)	0.50	1/13 (1.1)	1
Segmentation_hindbrain	67/159 (62.6)	1	*118/314 (87.7)*	*0.003*	6/61 (5.1)	1
Segmentation_hypochord	5/11 (4.3)	1	8/27 (7.5)	1	1/6 (0.5)	1
Segmentation_hypophysis	15/28 (11)	0.771	24/63 (17.6)	0.31	1/13 (1.1)	1
Segmentation_hypothalamus	14/35 (13.8)	1	35/87 (24.3)	0.09	0/17 (1.4)	1
Segmentation_immatureeye	24/74 (29.1)	0.853	33/139 (38.8)	0.65	0/40 (3.3)	0.82
Segmentation_innerear	4/13 (5.1)	1	6/24 (6.7)	1	0/5 (0.4)	1
Segmentation_intermediate cellmassofmesoderm	9/18 (7.1)	1	11/28 (7.8)	0.59	1/7 (0.6)	1
Segmentation_laterallinesystem	15/29 (11.4)	0.811	21/56 (15.6)	0.42	2/12 (1)	1
Segmentation_lateralplatemesoderm	0/8 (3.1)	0.548	1/12 (3.4)	0.58	0/6 (0.5)	1
Segmentation_medianfinfold	4/9 (3.5)	1	7/29 (8.1)	1	3/6 (0.5)	0.38
Segmentation_midbrain	43/112 (44.1)	1	68/228 (63.7)	0.97	1/45 (3.7)	1
Segmentation_midbrainhindbrainboundary	13/38 (15)	1	20/63 (17.6)	0.97	0/14 (1.2)	1
Segmentation_musculaturesystem	14/35 (13.8)	1	24/59 (16.5)	0.15	1/13 (1.1)	1
Segmentation_myotome	40/109 (42.9)	1	61/205 (57.3)	0.97	4/47 (3.9)	1
Segmentation_neuralcrest	12/26 (10.2)	1	21/50 (14)	0.15	0/11 (0.9)	1
Segmentation_neuralrod	9/18 (7.1)	1	14/32 (8.9)	0.24	1/7 (0.6)	1
Segmentation_neuraltube	33/74 (29.1)	1	49/152 (42.5)	0.59	4/27 (2.2)	1
Segmentation_neuron	37/79 (31.1)	0.773	50/133 (37.2)	0.09	6/32 (2.7)	0.75
Segmentation_olfactoryplacode	33/70 (27.6)	0.775	44/158 (44.1)	1	5/29 (2.4)	0.96
Segmentation_opticcup	7/8 (3.1)	0.232	8/17 (4.7)	0.41	0/1 (0.1)	1
Segmentation_opticvesicle	21/42 (16.5)	0.773	32/95 (26.5)	0.59	0/16 (1.3)	1
Segmentation_oticvesicle	32/82 (32.3)	1	51/190 (53.1)	1	3/41 (3.4)	1
Segmentation_pharyngealarch3-7	2/8 (3.1)	1	9/27 (7.5)	1	0/3 (0.2)	1
Segmentation_pharyngealarch3-7skeleton	20/55 (21.7)	1	30/109 (30.5)	1	3/32 (2.7)	1
Segmentation_pharyngealarch	25/51 (20.1)	0.773	46/117 (32.7)	0.06	3/23 (1.9)	1
Segmentation_pharynx	6/24 (9.4)	0.775	11/61 (17)	0.35	3/18 (1.5)	1
Segmentation_post-ventregion	4/5 (2)	0.738	4/9 (2.5)	0.86	0/0 (0)	1
Segmentation_posteriorcardinalvein	5/9 (3.5)	1	6/18 (5)	1	0/2 (0.2)	1
Segmentation_presumptivediencephalon	2/8 (3.1)	1	3/20 (5.6)	0.64	0/4 (0.3)	1
Segmentation_presumptiveneuralretina	1/3 (1.2)	1	2/9 (2.5)	1	0/2 (0.2)	1
Segmentation_primitivehearttube	8/20 (7.9)	1	11/33 (9.2)	0.97	1/10 (0.8)	1
Segmentation_pronephricduct	38/92 (36.2)	1	55/203 (56.7)	1	7/47 (3.9)	0.97
Segmentation_pronephros	8/22 (8.7)	1	14/56 (15.6)	1	0/11 (0.9)	1
Segmentation_rhombomere5	6/8 (3.1)	0.636	*12/20 (5.6)*	*0.03*	0/1 (0.1)	1
Segmentation_rhombomere	9/26 (10.2)	1	17/48 (13.4)	0.66	0/8 (0.7)	1
Segmentation_solidlensvesicle	31/59 (23.2)	0.548	*46/111 (31)*	*0.03*	4/29 (2.4)	1

Segmentation_somite	56/140 (55.1)	1	92/273 (76.3)	0.15	5/69 (5.7)	1
Segmentation_telencephalon	54/126 (49.6)	1	85/246 (68.7)	0.11	4/49 (4.1)	1
Segmentation_trigeminalganglion	12/19 (7.5)	0.548	*18/36 (10.1)*	*0.05*	2/4 (0.3)	0.75
Segmentation_trigeminalplacode	18/39 (15.4)	1	29/69 (19.3)	0.09	1/11 (0.9)	1
Segmentation_vein	9/22 (8.7)	1	18/40 (11.2)	0.12	1/9 (0.7)	1
Pharyngula_caudalfin	2/6 (2.4)	1	5/16 (4.5)	1	1/5 (0.4)	1
Pharyngula_epidermis	12/33 (13)	1	25/93 (26)	1	4/20 (1.7)	0.96
Pharyngula_eye	67/150 (59.1)	0.773	102/297 (83)	0.09	2/58 (4.8)	1
Pharyngula_heart	49/96 (37.8)	0.318	*72/191 (53.4)*	*0.03*	7/40 (3.3)	0.75
Pharyngula_hearttube	11/31 (12.2)	1	18/57 (15.9)	0.98	2/15 (1.2)	1
Pharyngula_intestinalbulb	7/30 (11.8)	0.674	16/75 (21)	0.59	1/19 (1.6)	1
Pharyngula_intestine	23/53 (20.9)	1	33/113 (31.6)	1	4/22 (1.8)	1
Pharyngula_lens	35/70 (27.6)	0.636	*66/145 (40.5)*	*<0.001*	5/33 (2.7)	1
Pharyngula_liver	53/129 (50.8)	1	86/308 (86.1)	1	7/61 (5.1)	1
Pharyngula_neuromast	6/22 (8.7)	0.977	10/51 (14.2)	0.59	1/12 (1)	1
Pharyngula_olfactorybulb	17/32 (12.6)	0.738	23/54 (15.1)	0.11	3/9 (0.7)	0.75
Pharyngula_olfactoryepithelium	*17/23 (9.1)*	*0.035*	24/54 (15.1)	0.08	2/5 (0.4)	0.78
Pharyngula_optictectum	54/129 (50.8)	1	72/235 (65.7)	0.78	6/54 (4.5)	1
Pharyngula_pancreaticbud	7/21 (8.3)	1	9/34 (9.5)	1	1/12 (1)	1
Pharyngula_pectoralfin	28/56 (22)	0.704	51/137 (38.3)	0.10	4/22 (1.8)	1
Pharyngula_pectoralfinbud	7/18 (7.1)	1	20/44 (12.3)	0.09	1/8 (0.7)	1
Pharyngula_pectoralfinmusculature	25/70 (27.6)	1	43/145 (40.5)	1	4/37 (3.1)	1
Pharyngula_peripheralolfactoryorgan	35/79 (31.1)	1	46/167 (46.7)	1	5/36 (3)	1
Pharyngula_retina	91/184 (72.4)	0.090	*123/341 (95.3)*	*0.01*	6/67 (5.6)	1
Pharyngula_retinalganglioncelllayer	*57/82 (32.3)*	*<0.001*	*73/131 (36.6)*	*<0.001*	4/17 (1.4)	0.75
Pharyngula_retinalinnernuclearlayer	*36/57 (22.4)*	*0.022*	*44/96 (26.8)*	*0.003*	2/15 (1.2)	1
Pharyngula_retinalphotoreceptorlayer	13/23 (9.1)	0.713	15/44 (12.3)	0.86	3/11 (0.9)	0.78
Pharyngula_spinalcord	59/127 (50)	0.674	89/263 (73.5)	0.15	6/46 (3.8)	1
Pharyngula_tegmentum	34/72 (28.3)	0.773	56/148 (41.4)	0.07	6/31 (2.6)	0.75
Pharyngula_ventricularzone	15/38 (15)	1	21/75 (21)	1	1/16 (1.3)	1
Hatching_ciliarymarginalzone	5/8 (3.1)	0.952	8/16 (4.5)	0.30	0/2 (0.2)	1
Hatching_habenula	12/23 (9.1)	0.878	16/35 (9.8)	0.13	1/7 (0.6)	1
Hatching_pancreas	6/15 (5.9)	1	10/33 (9.2)	1	1/9 (0.7)	1
Hatching_swimbladder	1/11 (4.3)	0.636	5/22 (6.1)	1	0/7 (0.6)	1
Larval_kidney	22/42 (16.5)	0.674	28/97 (27.1)	1	2/10 (0.8)	1
Larval_ovary	16/42 (16.5)	1	29/107 (29.9)	1	1/19 (1.6)	1
Larval_testis	23/54 (21.3)	1	38/115 (32.1)	0.59	2/21 (1.7)	1

a: In each of the three columns, three figures are given: first the number of ohnologs expressed in the tissue, given over the total number of ohnologs + single-copy genes expressed. In parentheses is then given the expected number of ohnologs, given their overall (non-zygotic) frequency.

b: Ohnologs preserved in all species verses single-copy genes in all species (*Methods*).
